# A comprehensive meta-analysis of common genetic variants in autism spectrum conditions

**DOI:** 10.1186/s13229-015-0041-0

**Published:** 2015-08-28

**Authors:** Varun Warrier, Vivienne Chee, Paula Smith, Bhismadev Chakrabarti, Simon Baron-Cohen

**Affiliations:** Autism Research Centre, Department of Psychiatry, University of Cambridge, Douglas House, 18B Trumpington Road, Cambridge, CB2 8AH UK; Centre for Integrative Neuroscience and Neurodynamics, School of Psychology and Clinical Language Sciences, University of Reading, Reading, UK; CLASS Clinic, Cambridgeshire and Peterborough NHS Foundation Trust (CPFT), Cambridgeshire, UK

**Keywords:** Meta-analysis, Association, Genetic variants, Insertions, Autism spectrum conditions

## Abstract

**Background:**

Autism spectrum conditions (ASC) are a group of neurodevelopmental conditions characterized by difficulties in social interaction and communication alongside repetitive and stereotyped behaviours. ASC are heritable, and common genetic variants contribute substantial phenotypic variability. More than 600 genes have been implicated in ASC to date. However, a comprehensive investigation of candidate gene association studies in ASC is lacking.

**Methods:**

In this study, we systematically reviewed the literature for association studies for 552 genes associated with ASC. We identified 58 common genetic variants in 27 genes that have been investigated in three or more independent cohorts and conducted a meta-analysis for 55 of these variants. We investigated publication bias and sensitivity and performed stratified analyses for a subset of these variants.

**Results:**

We identified 15 variants nominally significant for the mean effect size, 8 of which had *P* values below a threshold of significance of 0.01. Of these 15 variants, 11 were re-investigated for effect sizes and significance in the larger Psychiatric Genomics Consortium dataset, and none of them were significant. Effect direction for 8 of the 11 variants were concordant between both the datasets, although the correlation between the effect sizes from the two datasets was poor and non-significant.

**Conclusions:**

This is the first study to comprehensively examine common variants in candidate genes for ASC through meta-analysis. While for majority of the variants, the total sample size was above 500 cases and 500 controls, the total sample size was not large enough to accurately identify common variants that contribute to the aetiology of ASC.

**Electronic supplementary material:**

The online version of this article (doi:10.1186/s13229-015-0041-0) contains supplementary material, which is available to authorized users.

## Background

Autism spectrum conditions (ASC) are a group of neurodevelopmental conditions characterized by difficulties in social interaction and communication alongside unusually repetitive and stereotyped behaviour and unusually narrow interests [1]. ASC has an estimated heritability of around 50 % [2, 3], and common variants contribute to a significant proportion of the variability in the condition [3, 4]. ASC is polygenic and genetic variants, in addition to environmental, epigenetic and hormonal factors, contribute to ASC risk and phenotypic variability [5].

Sequencing and copy number variation analyses have identified a number of rare, highly penetrant, possibly causative variants. Strategies to identify common variants through genome-wide association studies have failed to produce consistent, replicable results across cohorts [5]. This may be attributed to many factors, including smaller than required sample size to adequately power these studies to identify variants with small effects. Over the last 15 years, a large number of studies have investigated common variants in candidate genes for ASC [6] typically investigating variants in a small number of genes using a relatively small sample size. These studies have provided some evidence of the association of a few genes with ASC, though they are not rigorous enough to definitively identify variants and results vary based on ethnicity, sample size, study methodology and clinical ascertainment [6]. One method to investigate the underlying effect using summary level data is meta-analysis [7]. Though not without limitations, meta-analysis provides a fairly robust statistical framework to systematically analyse effect sizes [7]. Further, the combined power of a meta-analysis greatly exceeds the power of the individual studies in a meta-analysis [7].

In the field of psychiatric genetics, studies have comprehensively investigated existing candidate gene studies and used meta-analysis to investigate genetic associations [8–10]. In the field of autism genetics, such an overarching study is lacking and no study, to our knowledge, has provided a comprehensive overview of ASC genetics. To bridge this gap, we reviewed the existing literature for 552 genes implicated in ASC. Using a strict inclusion criteria, we identified common variants in 27 genes that were investigated in three or more independent cohorts. We performed meta-analyses, sensitivity analyses and subgroup analyses for these common variants and checked for publication bias in a subset of these common variants. This is the first comprehensive study of candidate gene associations in ASC.

## Methods

### Literature search and inclusion criteria

A preliminary literature search of genes associated with ASC was performed using SFARI gene (https://gene.sfari.org/) and HuGE Navigator (http://hugenavigator.net/). Since both of these databases do not completely document the available literature, we additionally searched PubMed, Scopus and Google Scholar. The search terms used were ‘Gene name’ or ‘variant ID’ and ‘Autism’ or ‘Autistic Disorder’ or ‘Asperger Syndrome’.

Studies were included in the meta-analysis if: (1) they reported effect sizes or statistics to measure effect sizes and confidence intervals; (2) the studies were either a case-control association study or a transmission disequilibrium study of autism; (3) the variants did not deviate from Hardy-Weinberg Equilibrium (HWE) in the control group or if the sample size was too small to effectively calculate HWE due to sampling effect. Though we checked for HWE in family-based studies, this was not a requirement for including these studies as the study design overcomes the issue of population stratification; (4) cases had a diagnosis of an autism spectrum condition (Autism, PDDNOS, Asperger Syndrome) according to DSM-IV, DSM-5 or ICD-10 criteria; (5) the global minor allele frequency (MAF) of the variant investigated was greater than 0.01; (6) the studies were reported in English and (7) the common variants were investigated in independent cohorts. Authors of the articles were contacted if sufficient information was absent to use the data for meta-analysis. In addition to the published studies, we used unpublished genotype data from two cohorts from our research group at the Autism Research Centre, University of Cambridge. These cohorts are labelled ‘Chakrabarti [11]’ and ‘Warrier [12]’ in the current study. The characteristics of the two cohorts are described elsewhere [11, 12]. Details of genotyping and statistical analysis are provided in Additional file 1. We did not include data from genome-wide association studies (GWAS) as there is an overlap between participants in the candidate gene association studies and the genome-wide association studies. Since we had access to only summary data, it was impossible to ascertain the degree of overlap and remove participants accordingly. Literature search and study inclusion was performed independently by two researchers (VC and VW) from March 2014 to September 2014.

### Statistical analyses

Meta-analysis was performed only if variants were investigated in three or more independent cohorts. Family-based association tests (FBATs) studies were not included as effect sizes are not calculated in FBA. For variants investigated in five or more independent cohorts, we performed a complete meta-analysis. This included the calculation of effect size and publication bias, sensitivity analysis and subgroup analysis. For variants investigated in three to five independent cohorts, we performed a partial meta-analysis restricted to the calculation of mean effect size. We did not perform a meta-analysis for variants investigated in fewer than three cohorts as there was insufficient power to significantly investigate the underlying effect. For variants with *P* values <0.05 we calculated fail-safe N.

All analyses were performed using Comprehensive Meta-Analysis version 2.0 [13]. Meta-analysis was performed using the inverse-variance weighted method. Heterogeneity in the reported effects were examined using a fixed and a random effects model. Heterogeneity was measured using *I*^2^ statistics in conjunction with Q-statistics. A fixed effect model was applied if the *P* value for Q-statistics was above 0.05 and *I*^2^ was below 60. The random effects model was used if either the *P* value was below 0.05 or *I*^2^ was above 60, as an *I*^2^ above 60 indicates that 60 % of the total observed variation is due to true heterogeneity [7, 10].

Egger’s regression in conjunction with a funnel plot was used to assess publication bias. Sensitivity analyses were performed by removing each study from the meta-analysis and calculating the mean effect size for the remaining studies. This analysis was used to assess the contribution of each study to the final weighted effect in the analysis. Additionally, for the variants with *P* values <0.05, we computed both classic fail-safe N and Orwin’s fail-safe N to check the number of studies required to make the *P* value non-significant and make the effect size trivial respectively. For Orwin’s fail-safe N, the non-significant odds ratio (OR) was kept at 1.05 or 0.95 depending on the effect direction. While this is certainly not a trivial effect size, it is difficult to identify variants with such small effects with precision given the sample sizes in the meta-analysis. Subgroup analysis was performed after stratifying based on ethnicity or study methodology to check if either of these variables affected the final effect size. We conducted the subgroup analysis only for variants investigated in five or more independent cohorts. Meta-analysis was performed only if there were at least three independent cohorts after stratification to account for power considerations.

OR and 95 % confidence intervals (CI) were used to calculate the mean effect size. For transmission disequilibrium tests (TDT), odds ratios were calculated according to methods laid out by Kazeem and Farall [14]. Where possible, OR and CI were calculated using allele numbers for case-controls (CC) and transmitted and non-transmitted numbers for TDT. Where information of OR and CI was provided for the complement allele of the allele investigated in the study, the log odds ratio (LOR) and standard error (SE) were calculated and used in the meta-analysis.

Age was not regarded a confounding variable as ASC is a neurodevelopmental condition, and genetic variations are largely invariant across lifespan. However, ASC has a male-female ratio of 5:1 [5], and sex is a potential confounding variable as gene expressions can vary based on sex. However, there was insufficient data to conduct a stratified analysis based on sex, so this is a limitation of the current study. Finally, due to the large number of studies carried out, we adopted a more conservative statistical significance threshold of 0.01. This is similar to what was used in a similar comprehensive meta-analysis of obsessive-compulsive disorder [10]. We did not carry out a Bonferroni correction as the sample for each variant investigated was very different, and as a result, multiple tests were not carried out on the same sample.

### Analysis of the PGC dataset

While we did not choose to include data from available GWAS due to potential overlap of participants, we compared the results using the publicly available GWAS dataset from the Psychiatric Genomics Consortium (PGC). In the ASC cohort of the PGC dataset, 4788 trio cases and 4788 trio pseudocontrols as well as 161 cases and 526 controls have been genotyped. Details of the cohort, genotyping methods and statistical analysis are given elsewhere [15]. We searched for effect sizes and *P* values for variants with *P* values <0.05 in our meta-analysis. The autism PGC dataset is the largest available and accessible GWAS dataset for autism. The sample size of any of the variants investigated through meta-analysis in the study, except rs4141463 in *MACROD2*, is smaller than the sample size of the PGC autism dataset. Despite this, the PGC dataset is underpowered to detect variants with small effects. We were motivated to investigate the top variants in our study in the PGC dataset to ascertain if the candidate variants were at least nominally significant (*P* < 0.05) and if the effect direction was concordant between the two samples.

## Results

### Literature review

We identified 463 genes that have been tested for genetic association using HuGE Navigator (as of August 2014). SFARI Gene reports 616 genes to be associated with autism (as of August 2014). Only 185 of these genes have been examined in ASC using genetic association studies. Of these, we identified 89 genes from the SFARI Gene list that were not included in the HuGE Navigator list, bringing the total list of potential genes to 552. We did not identify any additional genes from AutismKB database. Thus, we reviewed 552 genes in total for the meta-analysis.

Scopus, Google Scholar and PubMed were searched for publications relating to ASC and any of the 552 genes. We searched for common variations in these genes that have been investigated for ASC in at least three independent cohorts. Using the eligibility criteria outlined in the methods section, we identified 27 genes that could be taken forward for meta-analysis. In total, there were 58 common variants across these 27 genes that were investigated in our meta-analysis. Details of the studies included and excluded for the 27 genes are given in Additional file 1: Tables S1 and S2.

**Fig. 1:**
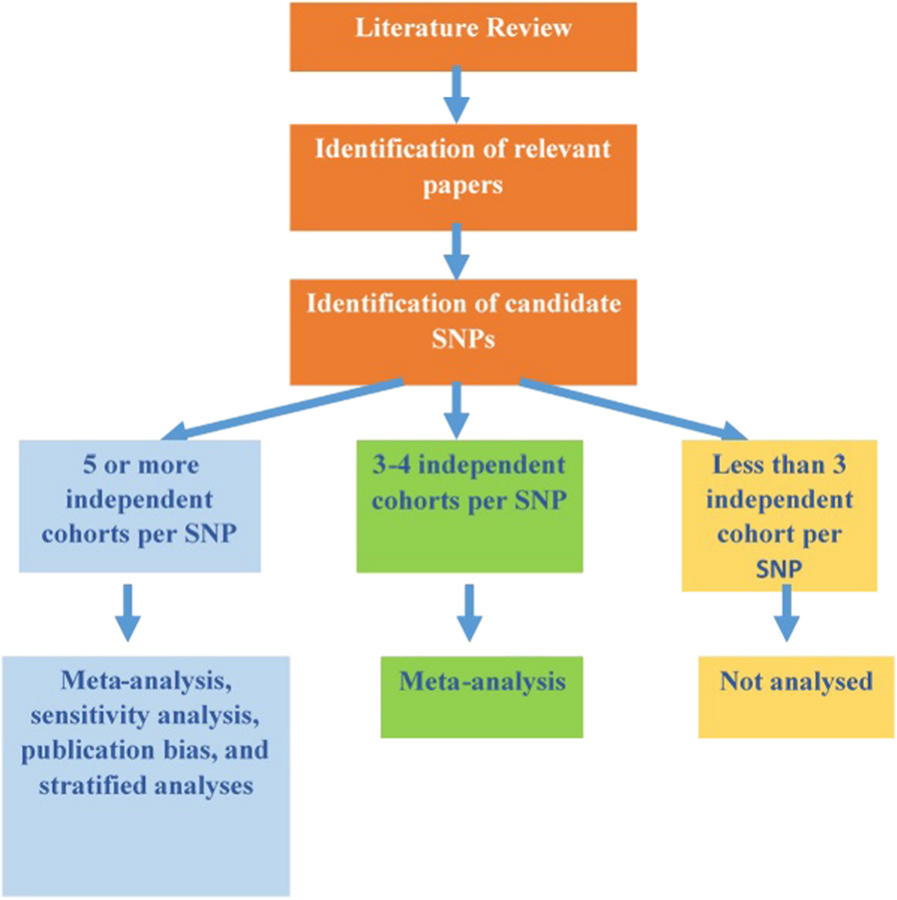
Schematic diagram of meta-analysis protocol

We next searched the literature for existing meta-analyses for the 58 variants and 27 genes in ASC, identifying existing meta-analyses for *OXTR* [16], *RELN* [17], *SLC6A4* [18], *HOXA1* [19], *HOXB1* [19] and *MTHFR* [20]*.* Detailed information of previous meta-analyses is provided in Additional file 1. As we had additional data and different inclusion criteria, we performed meta-analyses for all the variants in these six genes except rs723387731 in *HOXB1*, STin2 VNTR in *SLC6A4* and the GGC repeat in *RELN*. These three variants were excluded from the current meta-analyses as we could not identify additional data to add to the original meta-analyses. For the sake of comprehensiveness, we have included the data for these three variants in our table. Of the remaining 55 variants, we conducted a complete meta-analysis for 20 variants and a partial meta-analysis for 35 variants. A flow chart of the study protocol is given in Fig. 1.

### Mean effect sizes

Effect sizes for 15 variants in 12 genes had *P* values below 0.05. Nine of these variants had a *P* value below 0.01. The most significant association was rs167771 in *DRD3* (OR = 1.822, *P* value = 9.08 × 10^−6^). Seven other significant associations with *P* values <0.01 were in *CNTNAP2* (rs7794745, OR = 0.887, *P* value = 0.001), *RELN* (rs362691, OR = 0.832, *P* value = 3.93 × 10^−5^), *OXTR* (rs2268491, OR = 1.31, *P* value = 0.004), *SLC25A12* (rs2292813, OR = 1.372, *P* value = 0.001 and rs2056202, OR = 1.227, *P* value = 0.002), *EN2* (rs1861972, OR = 1.125, *P* value = 0.006) and *MTHFR* (rs1801133, OR = 1.370, *P* value = 0.010). As expected for common variants in ASC, the odds ratios for the alleles tested were small and lay between 0.781 (0.446–1.368) for *MAOA* uVNTR and 1.822 (1.398–2.375) for *DRD3* rs167771. Details of the variants analysed, model used and the *P* values are provided in Table 1. Forest plots for the nine most significant variants are in Additional file 1: Figures S1–S8.

**Table 1:** Summary of mean effect size analyses

S. No	Gene	Variants	Allele	Global MAF	Data sets	Mean OR (95% CI)	Z-Value	*P*-Value	Model (I2 value)	Total cases	Total controls	Trios	PGC *P*-value	Effect direction (odds ratio)	Classic fail-safe N	Orwin's fail safe N (OR = 1.05 or 0.95)
**1**	*DRD3*	**rs167771**	**G vs A**	**G=0.4113**	**3**	**1.822 (1.398-2.375)**	**4.44**	**9.08E-06**	**Fixed effect (60)**	**580**	**754**	**0**	**0.6**	**discordant (0.980)**	**7**	**34**
**2**	*RELN*	**rs362691**	**C vs G**	**C=0.1210**	**8**	**0.832 (0.763-0.908)**	**-4.11**	**3.93E-05**	**Fixed effect (33.2)**	**765**	**765**	**303**	**NA**	**NA**	**12**	**21**
**3**	*SLC25A12*	**rs2292813**	**C vs T**	**T=0.2085**	**6**	**1.372 (1.161-1.621)**	**3.72**	**1.97E-04**	**Fixed effect (0)**	**465**	**450**	**1220**	**0.78**	**concordant (1.014)**	**5**	**25**
**4**	*CNTNAP2*	**rs7794745**	**A vs T**	**A=0.4946**	**4**	**0.887 (0.828-0.950)**	**-3.45**	**1.00E-03**	**Fixed effect (21.2)**	**322**	**524**	**2236**	**0.18**	**concordant (0.9594)**	**9**	**6**
**5**	*SLC25A12*	**rs2056202**	**T vs C**	**T=0.2420**	**8**	**1.227 (1.079 -1.396)**	**3.12**	**2.00E-03**	**Fixed effect (6.5)**	**756**	**1211**	**1220**	**0.99**	**discordant (0.9993)**	**6**	**26**
**6**	*OXTR*	**rs2268491**	**T vs C**	**T=0.2137**	**4**	**1.31 (1.092 -1.572)**	**2.91**	**4.00E-03**	**Fixed effect (0)**	**282**	**440**	**458**	**0.54**	**concordant (1.026)**	**3**	**19**
**7**	*EN2*	**rs1861972**	**A vs G**	**G=0.242**	**8**	**1.125 (1.035-1.224)**	**2.75**	**6.00E-03**	**Fixed effect (57.6)**	**669**	**1704**	**953**	**NA**	**NA**	**16**	**12**
**8**	*MTHFR*	**rs1801133**	**T vs C**	**A=0.2454**	**10**	**1.370 (1.079-1.739)**	**2.59**	**1.00E-02**	**Random effects (88.2)**	**2280**	**7235**	**0**	**0.57**	**concordant (1.018)**	**80**	**40**
**9**	*ASMT*	**rs4446909**	**G vs A**	**A=0.1741**	**5**	**1.195 (1.038-1.375)**	**2.48**	**1.30E-02**	**Fixed effect (0)**	**1066**	**1074**	**0**	**NA**	**NA**	**3**	**14**
**10**	*MET*	**rs38845**	**A vs G**	**A=0.3634**	**3**	**1.322 (1.013-1.724)**	**2.41**	**1.60E-02**	**Random effects (66.5)**	**405**	**594**	**419**	**0.2**	**concordant (1.04)**	**13**	**15**
**11**	*SLC6A4*	**rs2020936**	**T vs C**	**G=0.228**	**4**	**1.244 (1.036-1.492)**	**2.35**	**1.90E-02**	**Fixed effect (33.9)**	**0**	**0**	**1068**	**0.78**	**concordant (1.01)**	**3**	**14**
**12**	*STX1A*	**rs4717806**	**A vs T**	**A=0.2322**	**4**	**0.851 (0.741-0.978)**	**-2.28**	**2.30E-02**	**Fixed effect (35.3)**	**653**	**1007**	**375**	**NA**	**NA**	**0**	**9**
**13**	*RELN*	**rs736707**	**T vs C**	**G=0.3660**	**9**	**1.269 (1.030-1.563)**	**2.24**	**2.50E-02**	**Random effects (76.5)**	**975**	**1695**	**196**	**0.31**	**concordant (1.035)**	**126**	**48**
**14**	*PON1*	**rs662**	**A vs G**	**T=0.4571**	**3**	**0.794 (0.642-0.983)**	**-2.12**	**3.40E-02**	**Fixed effect (17.5)**	**334**	**641**	**0**	**0.07**	**discordant (1.058)**	**0**	**11**
**15**	*OXTR*	**rs237887**	**G vs A**	**G=0.3998**	**4**	**1.163 (1.002-1.349)**	**1.99**	**4.70E-02**	**Fixed effect (0)**	**282**	**440**	**458**	**0.94**	**concordant (1.002)**	**0**	**9**
16	*STX1A*	rs6951030	G vs T	G=0.1771	4	1.383 (0.995-1.922)	1.93	5.40E-02	Random effects (76.7)	653	1007	375				
17	*OXTR*	rs2268493	C vs T	C=0.2049	3	0.845 (0.701-1.019)	-1.76	7.80E-02	Fixed effect (54.5)	574	1201	0				
18	*ASMT*	rs5989681	G vs C	NA	5	1.135 (0.984 - 1.308)	1.74	8.20E-02	Fixed effect (0)	1066	1074	0				
19	*HOXB1*	rs72338773*18	INS vs nINS	NA	8	1.36 (0.97-1.33)	NA	1.18E-01	Fixed Effect (NA)	362	448	238				
20	*RELN*	rs2073559	C vs T	C=0.4746	3	0.955 (0.900-1.014)	-1.5	1.35E-01	Fixed effect (64.5)	437	493	473				
21	*RELN*	GGC repeat*16	NA	NA	7	1.11 (0.80–1.54)	NA	1.53E-01	Fixed effect (0)	878	1170	167				
22	*GLO1*	rs2736654	A vs C	G=0.2873	4	1.307 (0.882 - 1.936)	1.34	1.82E-01	Random effects (68.7)	857	680	0				
23	*PON1*	rs854560	A vs T	T=0.1827	3	1.140 (0.931 - 1.395)	1.27	2.05E-01	Fixed effect (0)	334	641	0				
24	*TPH2*	rs11179000	T vs A	T=0.3988	3	1.130 (0.934-1.366)	1.26	2.08E-01	Fixed effect (0)	224	260	352				
25	*MET*	rs1858830	G vs C	G=0.4575	8	0.905 (0.773-1.061)	-1.23	2.19E-01	Random effect (67.5)	1975	1589	798				
26	*OXTR*	rs2268490	T vs C	T=0.2584	5	1.135 (0.920-1.400)	1.18	2.38E-01	Fixed effect (0)	292	761	458				
27	*OXTR*	rs2301261	A vs G	T=0.1248	4	1.127 (0.889-1.430)	0.99	3.22E-01	Fixed effect (39.1)	650	1300	0				
28	*HOXA1*	rs10951154	G vs A	C=0.2192	13	0.925 (0.791-1.081)	-0.98	3.28E-01	Fixed effect (35.7)	705	998	425				
29	*BDNF*	rs6265	G vs A	T=0.2013	3	0.919 (0.763-1.107)	-0.89	3.72E-01	Fixed effect (0)	303	469	140				
30	*HTR2A*	rs6311	A vs G	T=0.4435	6	0.871 (0.643-1.181)	-0.89	3.74E-01	Random effects (74.8)	179	313	396				
31	*ITGB3*	rs5918	C vs T	C=0.0889	3	0.866 (0.630-1.191)	-0.88	3.77E-01	Fixed effect (37.1)	139	165	363				
32	*MAOA*	uVNTR	short vs long	NA	3	0.781 (0.446 - 1.368)	-0.86	3.87E-01	Random effects (72)	436	469	0				
33	*MACROD2*	rs4141463	T vs C	C=0.3818	7	0.913 (0.734-1.135)	-0.82	4.11E-01	Random effects (87.1)	1170	35307	1158				
34	*OXTR*	rs2254298	A vs G	A=0.2071	5	0.813 (0.489-1.352)	-0.8	4.25E-01	Random effects (82.5)	650	1306	57				
35	*ASMT*	rs6644635	C vs T	NA	4	1.056 (0.906 -1.230)	0.69	4.88E-01	Fixed effect (29.3)	788	819	0				
36	*SLC6A4*	rs2020942	A vs G	T=0.2550	3	1.062 (0.881-1.281)	0.63	5.28E-01	Fixed effect (0)	0	0	678				
37	*OMG*	rs11080149	A vs G	T=0.0409	4	0.847 (0.477 - 1.506)	-0.56	5.72E-01	Random effects (43.8)	65	131	431				
38	*ADA*	rs7359837	G vs A	A=0.0282	3	1.375 (0.401 - 4.717)	0.51	6.13E-01	Random effects (89.1)	334	445	0				
39	*OXTR*	rs237894	G vs C	C=0.1615	5	0.961 (0.818-1.129)	-0.48	6.26E-01	Fixed effect (4)	292	761	458				
40	*OXTR*	rs53576	A vs G	A=0.3894	5	0.966 (0.839-1.113)	-0.48	6.31E-01	Fixed effect (44.9)	650	1300	57				
41	*OXTR*	rs2268494	A vs T	A=0.0683	4	1.076 (0.760 -1.510)	0.42	6.73E-01	Fixed effect (0)	76	99	458				
42	*SLC6A4*	STin2 VNTR*17	12 vs 9/10	NA	8	1.129 (0.819–1.558)	NA	6.73E-01	Random effects (68.7)	0	0	814				
43	*NF1*	GxAlu	9 vs non-9	NA	4	1.131 (0.633 - 2.022)	0.42	6.77E-01	Random effects (85.7)	262	312	0				
44	*GRIK2*	rs2227281	T vs C	T=0.2738	4	0.929 (0.603-1.432)	-0.34	7.32E-01	Random effects (77.3)	0	0	508				
45	*OXTR*	rs2268495	A vs G	A=0.2406	4	1.059 (0.763 - 1.468)	0.34	7.33E-01	Fixed effect (60.4)	282	446	458				
46	*SHANK3*	rs9616915	C vs T	C=0.3433	3	0.974 (0.834 - 1.138)	-0.33	7.44E-01	Fixed effect (60.1)	340	863	308				
47	*HTR2A*	rs6314	T vs G	A=0.0747	4	0.949 (0.691-1.304)	-0.32	7.47E-01	Fixed effect (18.3)	103	214	370				
48	*CNTNAP2*	rs2710102	T vs C	A=0.4113	3	0.989 (0.924-1.059)	-0.31	7.60E-01	Fixed effect (17.3)	322	524	2051				
49	*OXTR*	rs237885	G vs T	G=0.4884	6	0.981 (0.868 - 1.109)	-0.3	7.62E-01	Fixed effect (0)	574	1201	458				
50	*COMT*	rs4680	Met vs Val (A vs G)	A=0.3692	5	0.982 (0.851-1.134)	-0.24	8.08E-01	Fixed effect (49)	814	741	35				
51	*MTHFR*	rs1801131	C vs A	G=0.2494	6	0.979 (0.824-1.164)	-0.24	8.11E-01	Random effects (56.3)	1854	6819	0				
52	*OXTR*	rs1042778	G vs A	T=0.4109	4	1.02 (0.849-1.225)	0.21	8.33E-01	Fixed effect (0)	282	440	458				
53	*GRIK2*	rs2227283	A vs G	A=0.3275	4	0.967 (0.686-1.363)	-0.19	8.51E-01	Random effects (65.65)	0	0	508				
54	*EN2*	rs3735653	T vs C	T=0.4097	4	1.007 (0.870-1.165)	0.09	9.28E-01	Fixed effect (0)	174	349	499				
55	*NF1*	GxAlu	8 vs non-8	NA	4	0.982 (0.602 - 1.601)	-0.07	9.41E-01	Random effects (79.2)	262	312	0				
56	*SLC6A4*	5-HTTLPR	short vs long	NA	17	0.994 (0.847-1.167)	-0.07	9.42E-01	Random effects (63.8)	0	0	2039				
57	*HTR2A*	rs6313	T vs C	A=0.4413	3	1.007 (0.812-1.249)	0.07	9.47E-01	Fixed effect (0)	0	0	303				
58	*EN2*	rs1861973	T vs C	T=0.2410	6	1.004 (0.775-1.300)	0.03	9.77E-01	Random effects (80.8)	669	1704	681				

Rows highlighted in bold show variants with *P* values below 0.01

### Subgroup analyses

We performed subgroup analyses, stratifying by ethnicity and study methodology, for variants originally investigated in five or more independent cohorts. In the stratified analyses, six variants had *P* values below 0.05. Of these, the most significant three variants (rs2292813 and rs2056202-*SLC25A12*, rs362691-*RELN*) were also significant in the non-stratified analyses. Stratification did not increase the significance for these variants. A variant in *EN2* (rs1861973) was significant after stratifying based on both ethnicity (Caucasian only) and study methodology (TDT). Another variant in *EN2* (rs1861972) was significant after stratifying for study methodology (TDT). Finally, the STin2 variant in *SLC6A4* also exhibited a significant trend in the Caucasian-only subgroup. This result indicates that at least for a few variants implicated in ASC, ethnicity and study methodology can potentially influence the outcome. Results of the subgroup analyses are provided in Table 2. Forest plots for the significant and nominally significant subgroup analyses are provided in Additional file 1: Figures S9–S15.

**Table 2:** Summary of subgroup analyses

S.No	Gene	Variant	Allele	Data sets	Subgroup	Mean OR (95% CI)	Z-Value	*P*-Value	Model
1	*ASMT*	rs4446909	G vs A	3	Caucasian	1.135 (0.886 - 1.454)	1	3.16E-01	Fixed
2	*ASMT*	rs5989681	G vs C	3	Caucasian	1.065 (0.841 - 1.349)	0.52	6.03E-01	Fixed
3	*COMT*	rs4680	A vs G	4	TDT	0.973 (0.840 - 1.128)	−0.36	7.17E-01	Fixed
4	***EN2***	**rs1861973**	**T vs C**	**4**	**TDT**	**0.86 (0.791 - 0.954)**	**−2.94**	**3.00E-03**	**Fixed**
5	***EN2***	**rs1861973**	**T vs C**	**3**	**Caucasian**	**0.880 (0.801 - 0.969)**	**−2.26**	**9.00E-03**	**Fixed**
6	*EN2*	rs1861972	A vs G	4	Case–control	1.186 (0.876 - 1.605)	1.11	2.69E-01	Random
7	***EN2***	**rs1861972**	**A vs G**	**4**	**TDT**	**1.126 ( 1.025 - 1.238)**	**2.47**	**1.30E-02**	**Fixed**
8	*EN2*	rs1861972	A vs G	4	Caucasian	1.118 (0.807 - 1.549)	1.32	1.86E-01	Fixed
9	*HOXA1*	rs10951154	A vs G	6	Case–control	0.876 (0.675 - 1.137)	−0.99	3.21E-01	Random
10	*HOXA1*	rs10951154	A vs G	6	Caucasian	0.887 (0.661 - 1.190)	−0.8	4.23E-01	Random
11	*HOXA1*	rs10951154	A vs G	7	TDT	0.963 (0.784 - 1.159)	−0.48	6.32E-01	Random
12	*HTR2A*	rs6311	A vs G	4	TDT	0.893 (0.602 - 1.325)	−0.56	5.73E-01	Random
13	*HTR2A*	rs6311	A vs G	3	Caucasian	0.929 (0.542 - 1.594)	−0.27	7.90E-01	Random
14	*MACROD2*	rs4141463	T vs C	5	Case–control	1.033 (0.944 - 1.131)	0.71	4.78E-01	Random
15	*MET*	rs1858830	G vs C	7	Case–control	0.889 (0.749 - 1.056)	−1.34	1.80E-01	Random
16	*MET*	rs1858830	G vs C	3	Italian	0.924 (0.592 - 1.444)	−0.35	7.29E-01	Random
17	*MTHFR*	rs1801133	T vs C	4	Caucasian	1.398 (1.249 - 1.565)	5.82	6.60E-02	Random
18	*MTHFR*	rs1801131	C vs A	3	Caucasian	0.904 (0.782 - 1.044)	−1.37	1.71E-01	Fixed
19	*OXTR*	rs237885	G vs T	3	Case–control	0.950 (0.817 – 1.106)	−0.65	5.11E-01	Fixed
20	*OXTR*	rs2268490	T vs C	3	TDT	1.281 (0.953 - 1.721)	1.64	1.01E-01	Fixed
21	*OXTR*	rs2254298	A vs G	4	Caucasian	0.664 (0.357 - 1.235)	−1.29	1.96E-01	Fixed
22	*OXTR*	rs2268490	T vs C	4	Caucasian	1.114 (0.882 - 1.409)	0.91	3.66E-01	Fixed
23	*OXTR*	rs237885	G vs T	4	Caucasian	1.039 (0.885 - 1.220)	0.47	6.40E-01	Fixed
24	*OXTR*	rs237885	G vs T	3	TDT	1.043 (0.846 - 1.285)	0.39	6.96E-01	Fixed
25	*OXTR*	rs2254298	A vs G	4	Case–control	1.034 (0.693 - 1.542)	0.16	8.69E-01	Fixed
26	***RELN***	**rs362691**	**C vs G**	**6**	**Case–control**	**0.857 (0.783 - 0.939)**	**−3.32**	**1.00E-03**	**Fixed**
27	*RELN*	rs736707	T vs C	8	Case–control	1.187 (0.953 - 1.479)	1.53	1.27E-01	Random
28	*RELN*	rs736707	T vs C	3	Caucasian	1.307 (0.843 - 2.025)	1.2	2.32E-01	Random
29	***SLC25A12***	**rs2292813**	**C vs T**	**4**	**TDT**	**1.419 (1.158- 1.740)**	**3.52**	**7.33E-04**	**Fixed**
30	***SLC25A12***	**rs2056202**	**T vs C**	**5**	**TDT**	**1.275 (1.097 - 1.482)**	**3.17**	**2.00E-03**	**Fixed**
31	*SLC25A12*	rs2056202	T vs C	3	Case–control	1.105 (0.862 - 1.416)	0.79	4.31E-01	Fixed
32	*SLC25A12*	rs2056202	T vs C	4	Caucasian	1.087 (0.873 - 1.355)	0.75	4.55E-01	Fixed
33	*SLC6A4*	5-HTTLPR	short vs long	5	Caucasian	0.960 (0.650 - 1.418)	−0.2	8.38E-01	Fixed
34	***SLC6A4***	**STin2 VNTR**	**12 vs 9/10**	**4**	**Caucasian**	**1.492 (1.068 - 2.083)**	**2.34**	**1.90E-02**	**Fixed**

Rows highlighted in bold show variants with *P* values below 0.05

### Publication bias and sensitivity analyses

Publication bias was significant only for one variant, rs2254298 in *OXTR* (Egger’s test (two-tailed) *P* value = 0.03). However, the mean effect size for the variant was not significant (*P* value = 0.425). Notably, sensitivity was significant for some variants. Of the nine variants with *P* values below 0.01, we performed sensitivity analyses on the six variants with data from more than five independent cohorts (rs7794745, rs362691, rs2292813, rs2056202, rs1861972, and rs1801133). For rs1801133, most studies contributed approximately equally, with the exception of two studies [21, 22]; both of these studies lowered the OR. A re-analysis of the data after removing either of the two studies decreased the *P* value of the OR (original *P* value = 0.010, *P* value after removing Park et al., 2014 [21] = 0.006; *P* value after removing Schmidt et al., 2011 [22] = 0.003). For rs2056202, the removal of data from one study [23] increased the *P* value from *P* value = 0.002 to *P* value = 0.088. Sensitivity was not an issue for the remaining four variants that were significant. However, of the nominally significant variants, sensitivity was an issue for rs4446909, rs736707 and rs1861972. Forest graphs of the sensitivity analyses for these five variants are provided in Additional file 1: Figures S16–S20.

### Analysis of the PGC dataset

Of the 15 nominally significant variants in the current meta-analyses, 11 were genotyped in the PGC GWAS cohort, and none were found to be significant. Effect direction was concordant for 8 of the 11 variants between both the datasets. Effect sizes, as expected due to the larger sample size, were smaller in the PGC dataset for all the 11 variants, and the odds ratios were closer to 1. Total sample size was also not a significant predictor of concordance of effect direction between the two datasets. However, inspection of the datasets indicate that with the exception of rs2056202 in *SLC25A12*, the other three variants discordant for effect direction were analysed in small samples in the meta-analysis (see Table 2).

The lack of significance for 11 of the 15 variants in the PGC dataset forces us to re-evaluate the significance of the remaining four variants. For two variants, the classic fail-safe N is very small (three for rs4446909 in *ASMT*, and zero for rs4717806 in *STX1A*). The latter variant was analysed using a fixed effect model and becomes non-significant when analysed using a random effect model. For the remaining two variants (rs1861972 in *EN2* and rs362691 in *RELN*), the classic fail-safe N is above 10. The sample sizes, however, are modest. These analyses indicate that the first two variants are likely to be false positives. With rs1861972, the significance in *P* value is driven largely by the TDT-only subset in the original analysis (*P* value = 0.013, see Table 2). Both a case-control only subset and a Caucasian-only subset were not significant (see Table 2). rs1861972 is in high LD with rs1861973 (*r*^2^ = 1), and the two variants are separated by 152 base pairs. In this study, we used the random effects model to meta-analyse rs1861973 and it was not significant. Stratifying by both study methodology and ethnicity reduced the heterogeneity considerably, allowing us to use a fixed effect model. For rs1861973, both a Caucasian-only and a TDT-only subset were significant (see Table 2) but this variant was not significant in the larger Caucasian-only PGC cohort. Additional research in a larger, well-powered sample is required to confirm the significance of the two variants.

## Discussion

This is the first study to comprehensively investigate candidate gene association studies of common variants in ASC. Using two databases, we identified 552 genes that are reported to be implicated in ASC through genetic association studies. We scanned the literature for these 552 genes and, using a strict inclusion criteria, we identified 27 genes that had sufficient data to perform a meta-analysis. Eight variants across seven genes were significant for combined effect sizes with *P* values below 0.01. Data for 11 variants was present in the PGC GWAS dataset. None of the 11 variants were significant in the PGC dataset though the majority of the variants were concordant for effect direction in both the datasets.

Effect sizes for most common variants are modest for ASC, and these results are consistent with this observation. However, there was no clear correlation between effect sizes in our dataset and the PGC dataset. Effect sizes were smaller in the PGC dataset. While most of the effects lay between 0.8 and 1.2, which is expected from GWAS data, for some variants, the effect was larger. Our most significant variant (rs167771) had data only from three studies and had a relatively high OR of 1.82 to 1.40–2.38. The small sample size for this variant inflated the OR making it significant. The effect direction was discordant for the variant in the PGC dataset, and it was not significant in this dataset.

While the sample sizes for most variants were competitive for candidate gene association studies (above 500 total cases and 500 total controls), these are not sufficient to accurately calculate effect sizes. Additionally, the different study methodologies and ethnicities contributed to heterogeneity in the sample which potentially confounded the analyses. It is clear from this study that significant heterogeneity exists for a large fraction of the variants tested. In fact, heterogeneity is significantly and positively correlated with the number of independent datasets included per variant in the analyses, indicating that the current study may not have uncovered all the heterogeneity. We were able to remove some of the heterogeneity after stratifying for ethnicity and study methodology, but heterogeneity influenced the results for some for the variants even after this. This indicates that other additional factors contribute to variance in the effect. One potential source of heterogeneity is finer population stratification. Fine-scale population stratification cannot be addressed in candidate gene association studies as these test only a few variants. Further, HWE which is used to check for population admixture among other issues is performed individually for each variant in these studies thereby failing to utilize multi-marker information to correct for population stratification. We were unable to stratify based on sex or clinical ascertainment two factors known to contribute to heterogeneity in ASC. It is unclear how clinical heterogeneity maps onto genetic heterogeneity in ASC. Existing genetic studies that stratify based on IQ or other clinical phenotype and subphenotypes have had limited success [24, 25]. The inability to completely identify sources of heterogeneity forced us to choose between two models (fixed effect vs. random effects), when most variants are likely to have varying levels of heterogeneity. This is a significant concern for meta-analyses using candidate gene association studies. Even if sample sizes reach competitive levels, there are no techniques currently available that can accurately account for potential confounders such as ethnicity and study methodology. Both these issues can be satisfactorily addressed in GWAS.

Another cause for concern is the small number of genes with enough data to meta-analyse. Of 552 genes, we had data for only 27 of these, less than 5 %. None of the 27 genes analysed were ASC risk genes as predicted by DAWN [26]. Further, with the exception of *RELN* [27] and *SHANK3* [28], none of these genes have sufficient evidence to categorize them as risk genes using sequencing or copy number variation studies [27–31]. A few genes in the list of 552 genes but absent from the final list of 27 genes are predicted to be ASC risk genes. This includes *GABRB3*, *GRIN2B* and *SCN2A*. However, there was not enough evidence to evaluate the role of common variants in ASC for these genes through the current meta-analysis.

The majority of the studies analysed were of Caucasian ethnicity. We were able to stratify for a Caucasian ethnicity for some of the variants, but were not able to stratify for other ethnicities due to power considerations. It is also noteworthy that the PGC autism dataset used a Caucasian sample for analyses, and to our knowledge, there is no well-powered GWAS that investigates the role of common variants in autism in other ethnicities. Since the minor allele frequencies of the alleles tested and the variants tagged by these allele can vary depending on ethnicity, this makes it difficult to compare the results of the non-stratified meta-analyses with the PGC autism dataset. Replicating the top variants in well-powered samples from different ethnicities will help understand the ethnicity-specific risk for each variant.

The candidate gene association studies typically have small samples, which overestimate effect sizes. The lack of replication do not indicate that these loci do not contribute to the aetiology of ASC, but, rather, that there is insufficient evidence to implicate it in ASC. ASC is highly polygenic, and more than 49 % of its heritability can be attributed to common variants [3]. As effect size for each individual common variant are likely to be very modest and not likely to exceed an OR of 1.3, this indicates that there are several common variants that contribute to the condition. Disentangling this would require very large sample sizes, much larger than those in the current PGC autism GWAS. It is evident, from the current study, that candidate gene association studies in ASC have been underpowered to reliably detect causative variants with precision.

## Conclusions

While recent studies [2, 3] have identified that common variants, *en masse*, contribute to a significant fraction of ASC, there have not been any sufficiently powered studies to date to identify important common variants. We attempted to address this issue using a meta-analysis of candidate gene association studies. Though this is the first comprehensive study of candidate gene association studies in ASC, it failed to identify causative variants—11 of 15 variants with *P* values <0.05 were not significant in a larger sample from the PGC. Data was unavailable for the remaining five variants in the PGC dataset. We discuss the potential issues with such an approach and underline the need for much larger sample sizes to accurately identify common variants that contribute to ASC.

## **Additional file**

Additional file 1: Table S1. Studies included and study characteristics and** Table S2.** Studies Excluded; **Figures S1–S20.** Forest plots of significant variants from global and subgroup analysis, and sensitivity plots; details of data from our lab, details of previous meta-analyses, and references. (PDF 707 kb)

## Abbreviations

ASC: autism spectrum conditions
CC: case-control
CI: confidence intervals
FBAT: family-based association test
GWAS: genome-wide association study
HWE: Hardy-Weinberg Equilibrium
LOR: log odds ratio
MAF: minor allele frequency
OR: odds ratio
PGC: Psychiatric Genomics Consortium
SE: standard error
