# Supplementary material for: A comprehensive meta-analysis of common genetic variants in autism spectrum conditions
Source: Mol Autism. 2015 Aug 28;6:49. doi: 10.1186/s13229-015-0041-0 (PMC4552442; doi:10.1186/s13229-015-0041-0)
Supplement: Additional file 1: Table S1. — Studies included and study characteristics and Table S2. Studies Excluded; Figures S1–S20. Forest plots of significant variants from global and subgroup analysis, and sensitivity plots; details of data from our lab, details of previous meta-analyses, and references. (PDF 707 kb) [file 13229_2015_41_MOESM1_ESM.pdf]

Supplementary Material

Table of Contents

**Table 1: Studies included and study characteristics**	**Page 2**
**Table 2: Studies excluded**	**Page 20**
**References for the two table**	**Page 22**
**Supplementary Figures 1 – 8: Forest plots of most significant SNPs**	**Page 36**
**Supplementary Figures 9 – 15: Significant subgroup analyses**	**Page 44**
**Supplementary Figures 16 – 20: Sensitivity analyses forest plots**	**Page 51**
**Details of data from our lab**	**Page 56**
**Previous meta-analyses**	**Page 57**
**References**	**Page 58**

Table 1: Studies included and study characteristics

Gene	Study	Ancestry	Type of study	Sample size	SNPs included in the study	Diagnosis criteria						Hardy Weinberg Equilibrium	Notes
						ADOS	ADOS-G	ADI-R	CARS	DSM-IV	DSM III-R		
MTHFR	Park et al., 2014[1]	Korean	Case Control	251 cases, 425 controls	rs1801133					X		Yes	
	Liu et al., 2011[2]	Caucasian	Case Control	512 cases, 384 controls	rs1801133, rs1801131	X		X		X		Yes	Includes a proband from 205 simplex families and a random proband from 307 multiplex families
	Guo et al., 2012[3]	Chinese (Han)	Case Control	186 cases, 186 controls	rs1801133							Yes	
	dos Santos et al., 2010[4]	European derived (Brazil)	Case Control	151 cases, 100 controls	rs1801133			X				Yes	

	James et al., 2006[5]	Caucasian (97%)	Case Control	356 cases, 205 controls	rs1801133, rs1801131	X			X	X		Yes	
	Pasca et al., 2009[6]	Caucasian (Romanian)	Case Control	39 cases, 80 controls	rs1801133					X		Yes	
	Mohammad et al., 2009[7]	Indian	Case Control	138 cases, 138 controls	rs1801133					X		Yes	
	Divyakolu et al., 2013[8]	Indian	Case Control	50 cases, 50 controls	rs1801133							HWE not given, manually checked (Yes)	
	Boris et al., 2004[9]	Caucasian	Case Control	168 cases, 5389 controls	rs1801133, rs1801131					X		Yes	
	Schmidt et al., 2011[10]	Mixed	Case Control	429 cases, 278 controls	rs1801133	X		X				Yes	
EN2	Gharani et al., 2004[11]	Caucasian (AGRE sample)	Family based	167 families	rs1861972, rs1861973, rs3735653			X				Yes	
	Yang et al., 2010[12]	Chinese (Han)	Case Control	193 cases, 309 controls	rs1861972, rs1861973					X		Yes	
	Sen et al., 2010[13]	Indian	Family based	128 families of ASD children	rs1861973, rs3735653					X		Yes	

				comprising of 105 trios and 23 duos									
	Yang et al., 2008[14]	Chinese (Han)	Case Control	184 cases, 634 controls	rs1861972, rs1861973					X		Yes	Controls made of two groups, both the groups were combined in the analysis
	Prandini et al., 2008[15]	Italian	Family based	227 families	rs1861972						X		
	Benayed et al., 2005[16]	Caucasian (AGRE sample)	Family based	518 families	rs1861972, rs1861973			X				Yes	
	Warrier et al., 2014	Caucasian	Case Control	118 cases, 412 controls	rs1861972					X		Yes	
	Chakrabarti et al., 2009[17]	Caucasian	Case Control	174 cases, 349 controls	rs1861972, rs3735653					X		Yes	
	Zhong et al., 2003[18]	Caucasian (AGRE sample)	Family based	204 families	rs3735653			X			X	Yes	

GRIK2	Jamain et al., 2002[19]	European and American	Family based	107 trios	rs2227281, rs2227283			X		X		Yes	
	Dutta et al., 2007[20]	Indian	Family based	101 probands, 180 parents	rs2227281, rs2227283			X	X	X		Yes	
	Shuang et al., 2004[21]	Chinese (Han)	Family based	174 families	rs2227281, rs2227283					X			
	Kim et al., 2007[22]	Korean	Family based	126 trios	rs2227281, rs2227283	X		X		X		Yes	
COMT	Limprasert et al.,2014[23]	Thai	Family based	188 cases, 250 controls	Val158Met (rs4680)					X		Yes	Only Case-Control data used
	James et al., 2006[5]	Mixed	Case Control	360 cases and 205 controls	Val158Met (rs4680)			X	X	X		Yes	
	Guo et al., 2013[24]	Chinese Han	Case Control	186 cases, 186 controls	Val158Met (rs4680)				X	X		Yes	
	Karam et al., 2013[25]	Egyptian	Case Control	80 cases, 100 controls	Val158Met (rs4680)				X	X		Yes	

	Yirmiya et al., 2001[26]	N.A.	Family based	35 families	Val158Met (rs4680)			X		X	X	Yes	This study used haplotype relative risk and as a result, the data was treated as a case-control study
TPH2	Coon et al., 2005[27]	Mixed (Caucasian)	Case Control	88 cases, 95 controls	rs11179000	X		X				HWE not given, manually checked (Yes)	
	Ramoz et al., 2006[28]	Mixed	Family based	352 families	rs11179000			X				Yes	
	Singh et al., 2013[29]	Indian	Case Control	136 cases, 165 controls	rs11179000					X		Yes	
MACROD2	Curran et al., 2011[30]	Mixed	Case Control	1170 cases, 35307 controls	rs4141463			X		X		Yes	

	Prandini et al., 2008[15]	Italian	Family based	227 families	rs4141463						X	Yes	
	Anney et al., 2010[31]	Mixed	Family based	1158 families	rs4141463	X		X				Yes	
DRD3	Krom et al., 2009[32]	Dutch	Case Control	254 cases, 404 controls	rs167771					X		Yes	
	Toma et al., 2013[33]	Spanish	Case Control	326 cases, 350 controls	rs167771					X		Yes	
HTR2 A	Veenstra-VanderWeele et al., 2002[34]	Caucasian, African American, Asian American, Hispanic	Family based	115 trios	rs6311, rs6314					X		NA	
	Guhathakurta et al., 2009[35]	Indian	Family based and Case Control	97 trios	rs6311, rs6314					X		Yes	Only Family based data used
	Hranilovic et al., 2010[36]	Croation	Case Control	103 cases, 214 controls	rs6311, rs6314					X		Yes	
	Cho et al., 2007[37]	Korean	Family based	26 trios	rs6311				X			Yes	
	Smith et al., 2014[38]	Mixed	family based	158 trios	rs6311, rs6314	X				X		N.A.	
	Nyffeler et al., 2014[39]	Caucasian	Case Control	76 cases 99 controls	rs6311	X		X				Yes	

STX1A	Durdiaková et al., 2014[40]	Caucasian	Family based	479 cases, 650 controls	rs4717806, rs6951030					X		Yes	
	Nakamura et al., 2011[41]	Japanese	Family based	378 individuals	rs4717806, rs6951030			X		DSM-IV-TR		Yes	
	Nakamura et al., 2008[42]	Caucasian	Family based	249 trios	rs4717806, rs6951030	X		X				Yes	
	Chakrabarti et al., 2009[17]	Caucasian	Case Control	174 cases, 349 controls	rs4717806, rs6951030					X		Yes	
BDNF	Chakrabarti et al., 2009[17]	Caucasian	Case Control	174 cases, 349 controls	rs6265					X		Yes	
	Cheng et al., 2009[43]	Chinese	Case Control	174 cases, 349 controls	rs6265			X	X	X		Yes	
	Nishimura et al., 2007[44]	AGRE	Family based	104 trios	rs6265					X		Yes	
ITGB3	Singh et al., 2013[29]	Indian	Case Control	139 cases, 165 controls	rs5918				X	X		Yes	
	Cochrane et al., 2010[45]	Irish	Family based	177 trios	rs5918		X	X				Yes	
	Coutinho et al., 2007[46]	Portugese	Family based	186 trios	rs5918			X	X	X		NA	
CNTNAP2	Sampath et al., 2013[47]	Mixed	Family based	2051 families	rs7794745, rs2710102	X		X				Yes	
	Toma et al., 2013[48]	Spanish	Case Control	322 cases, 524 controls	rs7794745, rs2710102					X		Yes	

	Li et al., 2010[49]	Chinese	Family based	322 individuals	rs7794745					X		NA	
RELN	Sharma et al., 2013[50]	South African	Case Control	136 cases, 208 controls	rs736707, rs362691					X		Yes	
	Fu et al., 2013[51]	Chinese (Han)	Case Control	205 cases, 210 controls	rs2073559					X		Yes	
	He et al., 2011[52]	Chinese (Han)	Family based, Case Control	232 cases, 283 controls	rs736707, rs362691, rs2073559					X		NA	
	Dutta et al., 2008[53]	Indian	Family based, Case Control	102 cases, 283 controls	rs362691				X	X		Yes	
	Li et al., 2008[54]	Chinese (Han)	Case Control	213 cases, 160 controls	rs736707, rs362691					X		Yes	
	Bonora et al., 2003[55]	Mixed	Family based	342 cases, 194 controls	rs362691, GGC repeat	X		X				NA	
	Serajee et al., 2006[56]	Mixed	Family based	174 cases, 349 controls	rs736707, rs362691			X				Yes	
	Chakrabarti et al., 2009[17]	Caucasian	Case Control	174 cases, 349 controls	rs736707					X		Yes	
	Warrier et al., 2014	Caucasian	Case Control	118 cases, 412 controls	rs736707							Yes	

	Persico et al., 2001[57]	American/ Italian	Family based and Case Control	95 cases, 186 controls	GGC repeat					X		Yes	
	Krebs et al., 2002[58]	Mixed	Family based	167 families	GGC repeat			X		X		NA	
	Zhang et al., 2002[59]	Canada	Case Control	126 cases, 347 controls	GGC repeat	X		X				Yes	
	Li et al., 2004[60]	Mixed	Family based	107 families	GGC repeat	X		X				NA	
	Ashley-Koch et al., 2007[144]	Caucasian	Family based	470	rs2073559					X		Yes	
	Dutta et al., 2007[61]	Indian	Family based/Case Control	55 cases, 80 controls	rs736707, GGC repeat				X	X		Yes	
SLC25A12	Ramoz et al., 2004[62]	Egyptian	Family based	2000 (710,1280)	rs2056202	X				X		NA	
	Segurado et al., 2005[63]	Irish	Family based	158 trios	rs2292813, rs2056202					X		NA	
	Blasi et al., 2006[64]	Caucasian	Family based/Case Control	531 individuals (261, 174)	rs2056202					X		NA	
	Chien et al., 2010[65]	Chinese (Han)	Case Control	465 cases, 450 controls	rs2056202, rs2292813	X				X		Yes	
	Chakrabarti et al., 2009[17]	Caucasian	Case Control	174 cases, 349 controls	rs2056202					X		Yes	

	Correia et al., 2006[66]	Italian	Case Control	NA	rs2056202			X		X		Yes	
	Palmieri et al., 2010[67]	Caucasian	Family based	197 families	rs2056202, rs2292813							NA	
	Ramoz et al., 2008[68]	AJMGB	Family based	334 families	rs2292813	X				X		NA	
	Durdiakova et al., 2014[69]	Caucasian	Case Control	117 cases, 412 controls	rs2056202					X		Yes	
PON1	Pasca et al., 2010[70]	Romanians	Case Control	50 cases, 85 controls	rs662, rs854560					X		Yes	
	D'Amelio et al., 2005[71]	American caucasian/ Italians	Case Control/Family based	177 cases, 180 controls (Italians), 107 cases, 376 controls (Americans)	rs662, rs854560	X		X		X		Yes	Only Case Control data used
ASMT	Melke et al., 2008[72]	Caucasian	Case Control	278 cases, 255 controls	rs4446909, rs5989681			X		X		Yes	
	Toma et al., 2007[73]	Finnish, Italian and European (IMGSAC)	Case Control	127 cases, 100 controls (Finnish), 69 cases, 90 controls (Italian),	rs4446909, rs5989681, rs6644635	X		X		X		Yes	

				194 cases, 192 controls (European - IMGSAC)									
	Wang et al., 2013[74]	Chinese	Case Control	398 cases, 437 controls	rs4446909, rs5989 681, rs6644635				X	X	AB C	Yes	
ADA	Hettinger et al., 2008[75]	NA	Case Control	125 cases, 167 controls	rs7359837	X		X				Yes	
	Bottini et al., 2001[76]	Italian	Case Control	118 cases, 126 controls	rs7359837					X		Yes	
	Persico et al., 2000[77]	Italian	Case Control and Family based	91 cases, 152 controls	rs7359837					X		Yes	
SHAN K3	Sykes et al., 2009[78]	NA (IMGSAC cohort)	Family based and case-pseudocon trol	308 families	rs9616915		X					Yes	Case-pseudoc ontrol data was used for analysis
	Shao et al., 2014[79]	Chinese	Case Control	212 cases, 636 controls	rs9616915					X		Yes	

	Waga et al., 2011[80]	Japanese	Case Control	128 cases, 228 controls	rs9616915					X		HWE not given, manually checked (Yes)	
MAOA	Verma et al., 2014[81]	Indian	Case Control	194 cases, 227 controls	uvntr				X	X		Yes	
	Salem et al., 2013[82]	Egyptian	Case Control	53 cases, 30 controls	uvntr				X			Yes	
	Tassone et al., 2011[83]	NA	Case Control	189 cases, 167 controls	uvntr	X		X				Yes	
NF1	Marui et al., 2004[84]	Japanese	Case Control	74 cases, 122 controls	GxAlu -8 vs non-8 and 9 vs non-9					X		Yes	
	Mbarek et al., 1999[85]	NA	Case Control	85 cases, 90 controls	GxAlu -8 vs non-8 and 9 vs non-9					X		Yes	
	Plank et al., 2001[86]	Caucasian & African	Case Control	204 cases, 200 controls	GxAlu -8 vs non-8 and 9 vs non-9					X		Yes	
MET	Campbell et al., 2006[87]	Italian	Family based and Case Control	702 cases, 189 controls	rs1858830					X		Yes	Only Case Control data used

	Jackson et al., 2009[88]	South Carolina & Italian	Case Control	174 cases, 369 controls (South Carolina), 65 cases, 126 controls (Italian)	rs1858830	X		X	X			Yes	
	Sousa et al., 2009[89]	Caucasian & Italian	TDT	1621 caucasian, 84 italian trios	rs1858830, rs38845					X		Yes	
	Campbell et al., 2008[90]	Mixed, largely Caucasian	Case Control	629 cases, 312 controls	rs1858830					X		Yes	
	Thanseem et al., 2010[91]	Japanese	Family based	378 families	rs1858830, rs38845			X		X		Yes	
	Zhou et al., 2011[92]	Chinese	Case Control	405 cases, 594 controls	rs1858830, rs38845				X	X		Yes	
GLO1	Wu et al., 2008[93]	Chinese	Case Control	272 cases, 310 controls	rs2736654			X		X		Yes	
	Junaid et al., 2004[94]	Multi	Case Control	71 cases, 49 controls	rs2736654			X				Yes	
	Kovač et al., 2014[95]	Slovenian	Case Control	143 cases, 150 controls	rs2736654					X		Yes	

	Sacco et al., 2007[96]	Italian, Caucasian -American	Case Control	371 cases, 171 controls	rs2736654	X		X		X		Yes	
OXTR	Liu et al., 2010[97]	Japanese	Case Control	282 cases, 440 controls	rs2301261, rs2254298, rs2268495, rs2268491, rs237885, rs237887, rs53576, rs1042778, rs2268493					X		Yes	
	Jacob et al., 2007[98]	Caucasian	Family based	57 trios	rs2254298,rs53576	X		X		X		Yes	
	Tansey et al., 2010[99]	Caucasian	Family based	458 families	rs2268494, rs237894, rs2268495, rs2268490, rs2268491, rs237885, rs237887, rs1042778					X		NA	
	Chakrabarti et al., 2009[17]	Caucasian	Case Control	174 cases, 349 controls	rs2301261, rs2254298, rs237894, rs2268490, rs237885, rs53576, rs2268493					X		Yes	
	Nyffeler et al., 2014[39]	Caucasian	Case Control	76 cases 99 controls	rs2268494, rs2301261, rs2254298, rs53576	X		X				Yes	

	DiNapoli et al., 2014[100]	Caucasian	Case Control	118 cases, 412 controls	rs2301261, rs2254298, rs237894, rs2268490, rs237885, rs53576, rs2268493					X		Yes	
OMG	Vourc'h P et al., 2003[101]	Caucasian	Case Control	65 cases, 101 controls	rs11080149					X		Yes	
	Martin et al., 2007[102]	US, Canada, Italian	Family based	431 families	rs11080149		X	X		X		Yes	
HOXA 1	Chakrabarti et al., 2009[17]	Caucasian	Case Control	174 cases, 349 controls	rs10951154					X		Yes	
	Devlin et al., 2002[103]	Mixed	Family based	231 families	rs10951154	X		X		X		NA	

	Collins et al., 2003[104]	Mixed	Case Controls and Family based	204 cases, 159 controls in total; 187 families	rs10951154					X		Yes (Caucasian), No (African American)	We used Case Control for the caucasian population and Family based for the African-american population
	Conciatori et al., 2004[105]	Italian and Caucasian	Case Control and Family based	127 cases, 174 controls	rs10951154					X		No	Only Family based data used
	Sen et al., 2007[106]	Indian (Northern and Eastern)	Case Control	80 cases, 149 controls	rs10951154				X	X		Yes	
	Gallagher et al., 2004[107]	Irish	Family based	78 families	rs10951154	X		X				NA	

	Romano et al., 2003[108]	Italian	Family based and Case Control	85 cases, 132 controls	rs10951154					X		Yes	Only Case Control data used
	Talebizadeh et al., 2002[109]	Mixed	Case Control	35 cases, 35 controls	rs10951154				X	X		Yes	
	Li et al., 2002[110]	NA	Family based	110 multiplex	rs10951154	X		X				Yes	
	Ingram et al.,2000 [111]	Caucasian	Family based and Case Control	50 families	rs10951154					X		NA	
SLC6A 4	Ramoz et al., 2006[112]	AGRE	Family based	352 families	5-HTTLPR, rs2020936, rs2020942			X				NA	
	Devlin et et al., 2005[113]	NIH	Family based	390 families	5-HTTLPR, rs2020936			X		X		Yes	
	Kim et al., 2002[114]	Caucasian	Family based	115 trios	5-HTTLPR, VNTR	X		X		X		Yes	
	Cho et al., 2007[37]	Korean	Family based	126 trios	5-HTTLPR					X		Yes	
	Klauck et al., 1997[115]	Caucasian (One family: Asian)	Family based	65 trios	5-HTTLPR, VNTR	X		X		X		NA	
	Cook et al., 1997[116]	Caucasian, African-American, Hispanic-	Family based	86 families	5-HTTLPR, VNTR	X		X				NA	

		American, Asian-American											
	Conroy et al., 2004[117]	Irish	Family based	84 trios	5-HTTLPR, VNTR		X	X				Yes	
	Maestrini et al., 1999[118]	Caucasian	Family based	90 families	5-HTTLPR, VNTR		X					NA	
	Persico et al., 2000[119]	Italian/American	Family based	54 trios, 44 trios	5-HTTLPR					X		Yes	
	Tordjman et al., 2001[120]	Caucasian	Family based	71 trios	5-HTTLPR							NA	
	Yirmiya et al., 2001[26]	Isreal	Family based	34 families	5-HTTLPR			X		X	X	NA	
	Betancur et al., 2002[121]	Caucasian (Austria, Belgium, France, Italy, Norway, Sweden and United states)	Family based	53 families with 43 trios	5-HTTLPR, VNTR			X		X		NA	
	Coutinho et al., 2006[46]	Portugese	Family based	196 families	5-HTTLPR, VNTR			X	X	X		NA	
	Mulder et al., 2005[122]	Dutch	Family based	125 trios	5-HTTLPR, VNTR							NA	
	Koishi et al., 2006[123]	Japanese	Family based	104 trios	5-HTTLPR					X		Yes	
	Guhathakurta et al., 2006[124]	Indian	Family based	93 families	5-HTTLPR				X	X		Yes	
	Wu et al., 2005[125]	Chinese	Family based	175 trios	rs2020936, rs2020942			X		X		Yes	

	Yoo et al., 2009[126]	Korean	Family based	151 trios	rs2020936, rs2020942							Yes	

Table 2: Studies excluded

Study	Reason for exclusion	Article name
Alarcon et al. 2008[127]	Sample overlaps with Sampath *et al.* 2013	Linkage, association, and gene-expression analyses identify CNTNAP2 as an autism-susceptibility gene.
Anderson et al. 2008[128]	Insufficient data	Examination of association to autism of common genetic variation in genes related to dopamine.
Cheng et al. 2009[129]	Article inaccessible	Polyacrylamide gel-based microarray: a novel method applied to the association Study between the polymorphisms of BDNF gene and autism.
Egawa et al. 2012[130]	Minor allele frequency is 0	A detailed association analysis between the tryptophan hydroxylase 2 (TPH2) gene and autism spectrum disorders in a Japanese population.
Gaita et al. 2010[131]	Sample overlaps with D'amelio 2005	Decreased serum arylesterase activity in autism spectrum disorders
Hutcheson et al., 2004[132]	Insufficient data	Examination of NRCAM, LRRN3, KIAA0716, and LAMB1 as autism candidate genes
Kelemenova et al. 2010 [133]	Insufficient data	Polymorphisms of candidate genes in Slovak autistic patients.
Mei et al. 2007[134]	Covariates used in analysis	Multifactor dimensionality reduction-phenomics: a novel method to capture genetic heterogeneity with use of phenotypic variables.
Petit et al. 1995[135]	Insufficient data	Association study with two markers of a human homeogene in infantile autism.
Rabionet et al. 2006[136]	Insufficient data	Lack of association between autism and SLC25A12.
Rehnstrom et al. 2007[137]	Insufficient data	No association between common variants in glyoxalase 1 and autism spectrum disorders
Serajee et al. 2004[138]	Sample overlaps with D'amelio 2005	Polymorphisms in xeniobiotic metabolism genes and autism

Veatch et al. 2014[139]	Sample overlaps with Toma 2007, Melke 2008 and Wang 2013. Further tests specifically individuals with sleep issues.	Genetic Variation in Melatonin Pathway Enzymes in Children with Autism Spectrum Disorder and Comorbid Sleep Onset Delay
Weiss et al. 2006[140]	Tests for interaction	ITGB3 shows genetic and expression interaction with SLC6A4.
Xu et al. 2013[141]	Article inaccessible	Genetic polymorphisms of SNP loci in the 5' and 3' region of TPH2 gene in Northern Chinese Han population
McCauley et al., 2003[142]	Sample overlaps with Ramoz et al., 2006	Linkage and association analysis at the serotonin transporter (SLC6A4) locus in a rigid-compulsive subset of autism
Yu et al. 2004	Article inaccessible and not traceable	Association study between HOXA1 A218G polymorphism and autism.
Arking et al., 2008[143]	Sample overlaps with Sampath et al. 2013	A common genetic variant in the neurexin superfamily member CNTNAP2 increases familial risk of autism.

Studies mentioned in the table are studies that otherwise satisfy the inclusion criteria as mentioned in the Methods section. Several other studies were excluded as they did not meet all the criteria mentioned in the Methods section. These studies have not been listed in the table above.

References

1 Park J, Ro M, Pyun J-A, Nam M, Bang HJ, Yang JW et al. MTHFR 1298A>C is a risk factor for autism spectrum disorder in the Korean population. Psychiatry Res 2014; 215: 258–9.

2 Liu X, Solehdin F, Cohen IL, Gonzalez MG, Jenkins EC, Lewis MES et al. Population- and family-based studies associate the MTHFR gene with idiopathic autism in simplex families. J Autism Dev Disord 2011; 41: 938–44.

3 Guo T, Chen H, Liu B, Ji W, Yang C. Methylenetetrahydrofolate reductase polymorphisms C677T and risk of autism in the Chinese Han population. Genet Test Mol Biomarkers 2012; 16: 968–73.

4 Dos Santos PAC, Longo D, Brandalize APC, Schüler-Faccini L. MTHFR C677T is not a risk factor for autism spectrum disorders in South Brazil. Psychiatr Genet 2010; 20: 187–9.

5 James SJ, Melnyk S, Jernigan S, Cleves MA, Halsted CH, Wong DH et al. Metabolic endophenotype and related genotypes are associated with oxidative stress in children with autism. Am J Med Genet B Neuropsychiatr Genet 2006; 141B: 947–56.

6 Paşca SP, Dronca E, Kaucsár T, Craciun EC, Endreffy E, Ferencz BK et al. One carbon metabolism disturbances and the C677T MTHFR gene polymorphism in children with autism spectrum disorders. J Cell Mol Med 2009; 13: 4229–38.

7 Mohammad NS, Jain JMN, Chintakindi KP, Singh RP, Naik U, Akella RRD. Aberrations in folate metabolic pathway and altered susceptibility to autism. Psychiatr Genet 2009; 19: 171–6.

8 Divyakolu S, Tejaswini Y, Thomas W, Thumoju S, Sreekanth VR, Vasavi M et al. Evaluation of C677T Polymorphism of the Methylenetetrahydrofolate Reductase (MTHFR) Gene in various Neurological Disorders. J Neurol Disord 2013; 2:142

9 Boris M, Goldblatt A, Galanko J, James J. Association of MTHFR Gene Variants with Autism. J. Am. Physicians Surg. 2004; : 106 – 108.

10 Schmidt RJ, Hansen RL, Hartiala J, Allayee H, Schmidt LC, Tancredi DJ et al. Prenatal vitamins, one-carbon metabolism gene variants, and risk for autism. Epidemiology 2011; 22: 476–85.

11 Gharani N, Benayed R, Mancuso V, Brzustowicz LM, Millonig JH. Association of the homeobox transcription factor, ENGRAILED 2, 3, with autism spectrum disorder. Mol Psychiatry 2004; 9: 474–84.

12 Yang P, Shu B-C, Hallmayer JF, Lung F-W. Intronic single nucleotide polymorphisms of engrailed homeobox 2 modulate the disease vulnerability of autism in a han chinese population. Neuropsychobiology 2010; 62: 104–15.

13 Sen B, Singh AS, Sinha S, Chatterjee A, Ahmed S, Ghosh S et al. Family-based studies indicate association of Engrailed 2 gene with autism in an Indian population. Genes Brain Behav 2010; 9: 248–55.

14 Wang L, Jia M, Yue W, Tang F, Qu M, Ruan Y et al. Association of the ENGRAILED 2 (EN2) gene with autism in Chinese Han population. Am J Med Genet B Neuropsychiatr Genet 2008; 147B: 434–8.

15 Prandini P, Pasquali A, Malerba G, Marostica A, Zusi C, Xumerle L et al. The association of rs4307059 and rs35678 markers with autism spectrum disorders is replicated in Italian families. Psychiatr Genet 2012; 22: 177–81.

16 Benayed R, Gharani N, Rossman I, Mancuso V, Lazar G, Kamdar S et al. Support for the homeobox transcription factor gene ENGRAILED 2 as an autism spectrum disorder susceptibility locus. Am J Hum Genet 2005; 77: 851–68.

17 Chakrabarti B, Dudbridge F, Kent L, Wheelwright S, Hill-Cawthorne G, Allison C et al. Genes related to sex steroids, neural growth, and social-emotional behavior are associated with autistic traits, empathy, and Asperger syndrome. Autism Res 2009; 2: 157–77.

18 Zhong H, Serajee FJ, Nabi R, Huq AHMM. No association between the EN2 gene and autistic disorder. J Med Genet 2003; 40: e4.

19 Jamain S, Betancur C, Quach H, Philippe A, Fellous M, Giros B et al. Linkage and association of the glutamate receptor 6 gene with autism. Mol Psychiatry 2002; 7: 302–10.

20 Dutta S, Das S, Guhathakurta S, Sen B, Sinha S, Chatterjee A et al. Glutamate receptor 6 gene (GluR6 or GRIK2) polymorphisms in the Indian population: a genetic association study on autism spectrum disorder. Cell Mol Neurobiol 2007; 27: 1035–47.

21 Shuang M, Liu J, Jia MX, Yang JZ, Wu SP, Gong XH et al. Family-based association study between autism and glutamate receptor 6 gene in Chinese Han trios. Am J Med Genet B Neuropsychiatr Genet 2004; 131B: 48–50.

22 Kim SA, Kim JH, Park M, Cho IH, Yoo HJ. Family-based association study between GRIK2 polymorphisms and autism spectrum disorders in the Korean trios. Neurosci Res 2007; 58: 332–5.

23 Limprasert P, Maisrikhaw W, Sripo T, Wirojanan J, Hansakunachai T, Roongpraiwan R et al. No association of Val158Met variant in the COMT gene with autism spectrum disorder in Thai children. Psychiatr Genet 2014; 24: 230–1.

24 Guo T, Wang W, Liu B, Chen H, Yang C. Catechol-O-methyltransferase Val158Met polymorphism and risk of autism spectrum disorders. J Int Med Res 2013; 41: 725–34.

25 Karam RA, Rezk NA, Abdelrahman HM, Hassan TH, Mohammad D, Hashim HM et al. Catechol-O-methyltransferase Val158Met polymorphism and hyperactivity symptoms in Egyptian children with autism spectrum disorder. Res Dev Disabil 2013; 34: 2092–7.

26 Yirmiya N, Pilowsky T, Nemanov L, Arbelle S, Feinsilver T, Fried I et al. Evidence for an association with the serotonin transporter promoter region polymorphism and autism. Am J Med Genet 2001; 105: 381–6.

27 Coon H, Dunn D, Lainhart J, Miller J, Hamil C, Battaglia A et al. Possible association between autism and variants in the brain-expressed tryptophan hydroxylase gene (TPH2). Am J Med Genet B Neuropsychiatr Genet 2005; 135B: 42–6.

28 Ramoz N, Cai G, Reichert JG, Corwin TE, Kryzak LA, Smith CJ et al. Family-based association study of TPH1 and TPH2 polymorphisms in autism. Am J Med Genet B Neuropsychiatr Genet 2006; 141B: 861–7.

29 Singh AS, Chandra R, Guhathakurta S, Sinha S, Chatterjee A, Ahmed S et al. Genetic association and gene-gene interaction analyses suggest likely involvement of ITGB3 and TPH2 with autism spectrum disorder (ASD) in the Indian population. Prog Neuropsychopharmacol Biol Psychiatry 2013; 45: 131–43.

30 Curran S, Bolton P, Rozsnyai K, Chiocchetti A, Klauck SM, Duketis E et al. No association between a common single nucleotide polymorphism, rs4141463, in the MACROD2 gene and autism spectrum disorder. Am J Med Genet B Neuropsychiatr Genet 2011; 156B: 633–9.

31 Anney R, Klei L, Pinto D, Regan R, Conroy J, Magalhaes TR et al. A genome-wide scan for common alleles affecting risk for autism. Hum Mol Genet 2010; 19: 4072–82.

32 De Krom M, Staal WG, Ophoff RA, Hendriks J, Buitelaar J, Franke B et al. A common variant in DRD3 receptor is associated with autism spectrum disorder. Biol Psychiatry 2009; 65: 625–30.

33 Toma C, Hervás A, Balmaña N, Salgado M, Maristany M, Vilella E et al. Neurotransmitter systems and neurotrophic factors in autism: association study of 37 genes suggests involvement of DDC. World J Biol Psychiatry 2013; 14: 516–27.

34 Veenstra-VanderWeele J, Kim S-J, Lord C, Courchesne R, Akshoomoff N, Leventhal BL et al. Transmission disequilibrium studies of the serotonin 5-HT2A receptor gene (HTR2A) in autism. Am J Med Genet 2002; 114: 277–83.

35 Guhathakurta S, Singh AS, Sinha S, Chatterjee A, Ahmed S, Ghosh S et al. Analysis of serotonin receptor 2A gene (HTR2A): association study with autism spectrum disorder in the Indian population and investigation of the gene expression in peripheral blood leukocytes. Neurochem Int 2009; 55: 754–9.

36 Hranilovic D, Blazevic S, Babic M, Smurinic M, Bujas-Petkovic Z, Jernej B. 5-HT2A receptor gene polymorphisms in Croatian subjects with autistic disorder. Psychiatry Res 2010; 178: 556–8.

37 Cho IH, Yoo HJ, Park M, Lee YS, Kim SA. Family-based association study of 5-HTTLPR and the 5-HT2A receptor gene polymorphisms with autism spectrum disorder in Korean trios. Brain Res 2007; 1139: 34–41.

38 Smith RM, Banks W, Hansen E, Sadee W, Herman GE. Family-based clinical associations and functional characterization of the serotonin 2A receptor gene (HTR2A) in autism spectrum disorder. Autism Res 2014; 7: 459–67.

39 Nyffeler J, Walitza S, Bobrowski E, Gundelfinger R, Grünblatt E. Association study in siblings and case-controls of serotonin- and oxytocin-related genes with high functioning autism. J Mol Psychiatry 2014; 2: 1.

40 Durdiaková J, Warrier V, Banerjee-Basu S, Baron-Cohen S, Chakrabarti B. STX1A and Asperger syndrome: a replication study. Mol Autism 2014; 5: 14.

41 Nakamura K, Iwata Y, Anitha A, Miyachi T, Toyota T, Yamada S et al. Replication study of Japanese cohorts supports the role of STX1A in autism susceptibility. Prog Neuropsychopharmacol Biol Psychiatry 2011; 35: 454–8.

42 Nakamura K, Anitha A, Yamada K, Tsujii M, Iwayama Y, Hattori E et al. Genetic and expression analyses reveal elevated expression of syntaxin 1A (STX1A) in high functioning autism. Int J Neuropsychopharmacol 2008; 11: 1073–84.

43 Cheng L, Ge Q, Xiao P, Sun B, Ke X, Bai Y et al. Association study between BDNF gene polymorphisms and autism by three-dimensional gel-based microarray. Int J Mol Sci 2009; 10: 2487–500.

44 Nishimura K, Nakamura K, Anitha A, Yamada K, Tsujii M, Iwayama Y et al. Genetic analyses of the brain-derived neurotrophic factor (BDNF) gene in autism. Biochem Biophys Res Commun 2007; 356: 200–6.

45 Cochrane LE, Tansey KE, Gill M, Gallagher L, Anney RJL. Lack of association between markers in the ITGA3, ITGAV, ITGA6 and ITGB3 and autism in an Irish sample. Autism Res 2010; 3: 342–4.

46 Coutinho AM, Sousa I, Martins M, Correia C, Morgadinho T, Bento C et al. Evidence for epistasis between SLC6A4 and ITGB3 in autism etiology and in the determination of platelet serotonin levels. Hum Genet 2007; 121: 243–56.

47 Sampath S, Bhat S, Gupta S, O’Connor A, West AB, Arking DE et al. Defining the contribution of CNTNAP2 to autism susceptibility. PLoS One 2013; 8: e77906.

48 Toma C, Hervás A, Torrico B, Balmaña N, Salgado M, Maristany M et al. Analysis of two language-related genes in autism: a case-control association study of FOXP2 and CNTNAP2. Psychiatr Genet 2013; 23: 82–5.

49 Li X, Hu Z, He Y, Xiong Z, Long Z, Peng Y et al. Association analysis of CNTNAP2 polymorphisms with autism in the Chinese Han population. Psychiatr Genet 2010; 20: 113–7.

50 Sharma JR, Arieff Z, Gameeldien H, Davids M, Kaur M, van der Merwe L. Association analysis of two single-nucleotide polymorphisms of the RELN gene with autism in the South African population. Genet Test Mol Biomarkers 2013; 17: 93–8.

51 Fu X, Mei Z, Sun L. Association between the g.296596G > A genetic variant of RELN gene and susceptibility to autism in a Chinese Han population. Genet Mol Biol 2013; 36: 486–9.

52 He Y, Xun G, Xia K, Hu Z, Lv L, Deng Z et al. No significant association between RELN polymorphism and autism in case-control and family-based association study in Chinese Han population. Psychiatry Res 2011; 187: 462–4.

53 Dutta S, Sinha S, Ghosh S, Chatterjee A, Ahmed S, Usha R. Genetic analysis of reelin gene (RELN) SNPs: no association with autism spectrum disorder in the Indian population. Neurosci Lett 2008; 441: 56–60.

54 Li H, Li Y, Shao J, Li R, Qin Y, Xie C et al. The association analysis of RELN and GRM8 genes with autistic spectrum disorder in Chinese Han population. Am J Med Genet B Neuropsychiatr Genet 2008; 147B: 194–200.

55 Bonora E, Beyer KS, Lamb JA, Parr JR, Klauck SM, Benner A et al. Analysis of reelin as a candidate gene for autism. Mol Psychiatry 2003; 8: 885–92.

56 Serajee FJ, Zhong H, Mahbubul Huq AHM. Association of Reelin gene polymorphisms with autism. Genomics 2006; 87: 75–83.

57 Persico AM, D'Agruma L, Maiorano N, Totaro A, Militerni R, Bravaccio C et al. Reelin gene alleles and haplotypes as a factor predisposing to autistic disorder. Mol Psychiatry 2001; 6: 150–9.

58 Krebs MO, Betancur C, Leroy S, Bourdel MC, Gillberg C, Leboyer M. Absence of association between a polymorphic GGC repeat in the 5' untranslated region of the reelin gene and autism. Mol Psychiatry 2002; 7: 801–4.

59 Zhang H, Liu X, Zhang C, Mundo E, Macciardi F, Grayson DR et al. Reelin gene alleles and susceptibility to autism spectrum disorders. Mol Psychiatry 2002; 7: 1012–7.

60 Li J, Nguyen L, Gleason C, Lotspeich L, Spiker D, Risch N et al. Lack of evidence for an association between WNT2 and RELN polymorphisms and autism. Am J Med Genet B Neuropsychiatr Genet 2004; 126B: 51–7.

61 Dutta S, Guhathakurta S, Sinha S, Chatterjee A, Ahmed S, Ghosh S et al. Reelin gene polymorphisms in the Indian population: a possible paternal 5'UTR-CGG-repeat-allele effect on autism. Am J Med Genet B Neuropsychiatr Genet 2007; 144B: 106–12.

62 Ramoz N. Linkage and Association of the Mitochondrial Aspartate/Glutamate Carrier SLC25A12 Gene With Autism. Am J Psychiatry 2004; 161: 662–669.

63 Segurado R, Conroy J, Meally E, Fitzgerald M, Gill M, Gallagher L. Confirmation of association between autism and the mitochondrial aspartate/glutamate carrier SLC25A12 gene on chromosome 2q31. Am J Psychiatry 2005; 162: 2182–4.

64 Blasi F, Bacchelli E, Carone S, Toma C, Monaco AP, Bailey AJ et al. SLC25A12 and CMYA3 gene variants are not associated with autism in the IMGSAC multiplex family sample. Eur J Hum Genet 2006; 14: 123–6.

65 Chien W-H, Wu Y-Y, Gau SS-F, Huang Y-S, Soong W-T, Chiu Y-N et al. Association study of the SLC25A12 gene and autism in Han Chinese in Taiwan. Prog Neuropsychopharmacol Biol Psychiatry 2010; 34: 189–92.

66 Correia C, Coutinho AM, Diogo L, Grazina M, Marques C, Miguel T et al. Brief report: High frequency of biochemical markers for mitochondrial dysfunction in autism: no association with the mitochondrial aspartate/glutamate carrier SLC25A12 gene. J Autism Dev Disord 2006; 36: 1137–40.

67 Palmieri L, Papaleo V, Porcelli V, Scarcia P, Gaita L, Sacco R et al. Altered calcium homeostasis in autism-spectrum disorders: evidence from biochemical and genetic studies of the mitochondrial aspartate/glutamate carrier AGC1. Mol Psychiatry 2010; 15: 38–52.

68 Ramoz N, Cai G, Reichert JG, Silverman JM, Buxbaum JD. An analysis of candidate autism loci on chromosome 2q24-q33: evidence for association to the STK39 gene. Am J Med Genet B Neuropsychiatr Genet 2008; 147B: 1152–8.

69 Durdiaková J, Warrier V, Baron-Cohen S, Chakrabarti B. Single nucleotide polymorphism rs6716901 in SLC25A12 gene is associated with Asperger syndrome. Mol Autism 2014; 5: 25.

70 Paşca SP, Dronca E, Nemeş B, Kaucsár T, Endreffy E, Iftene F et al. Paraoxonase 1 activities and polymorphisms in autism spectrum disorders. J Cell Mol Med 2010; 14: 600–7.

71 D’Amelio M, Ricci I, Sacco R, Liu X, D’Agruma L, Muscarella LA et al. Paraoxonase gene variants are associated with autism in North America, but not in Italy: possible regional specificity in gene-environment interactions. Mol Psychiatry 2005; 10: 1006–16.

72 Melke J, Goubran Botros H, Chaste P, Betancur C, Nygren G, Anckarsäter H et al. Abnormal melatonin synthesis in autism spectrum disorders. Mol Psychiatry 2008; 13: 90–8.

73 Toma C, Rossi M, Sousa I, Blasi F, Bacchelli E, Alen R et al. Is ASMT a susceptibility gene for autism spectrum disorders? A replication study in European populations. Mol Psychiatry 2007; 12: 977–9.

74 Wang L, Li J, Ruan Y, Lu T, Liu C, Jia M et al. Sequencing ASMT identifies rare mutations in Chinese Han patients with autism. PLoS One 2013; 8: e53727.

75 Hettinger JA, Liu X, Holden JJA. The G22A polymorphism of the ADA gene and susceptibility to autism spectrum disorders. J Autism Dev Disord 2008; 38: 14–9.

76 Bottini N, De Luca D, Saccucci P, Fiumara A, Elia M, Porfirio MC et al. Autism: evidence of association with adenosine deaminase genetic polymorphism. Neurogenetics 2001; 3: 111–3.

77 Persico AM, Militerni R, Bravaccio C, Schneider C, Melmed R, Trillo S et al. Adenosine deaminase alleles and autistic disorder: case-control and family-based association studies. Am J Med Genet 2000; 96: 784–90.

78 Sykes NH, Toma C, Wilson N, Volpi E V, Sousa I, Pagnamenta AT et al. Copy number variation and association analysis of SHANK3 as a candidate gene for autism in the IMGSAC collection. Eur J Hum Genet 2009; 17: 1347–53.

79 Shao S, Xu S, Yang J, Zhang T, He Z, Sun Z et al. A commonly carried genetic variant, rs9616915, in SHANK3 gene is associated with a reduced risk of autism spectrum disorder: replication in a Chinese population. Mol Biol Rep 2014; 41: 1591–5.

80 Waga C, Okamoto N, Ondo Y, Fukumura-Kato R, Goto Y-I, Kohsaka S et al. Novel variants of the SHANK3 gene in Japanese autistic patients with severe delayed speech development. Psychiatr Genet 2011; 21: 208–11.

81 Verma D, Chakraborti B, Karmakar A, Bandyopadhyay T, Singh AS, Sinha S et al. Sexual dimorphic effect in the genetic association of monoamine oxidase A (MAOA) markers with autism spectrum disorder. Prog Neuropsychopharmacol Biol Psychiatry 2014; 50: 11–20.

82 Salem AM, Ismail S, Zarouk WA, Abdul Baky O, Sayed AA, Abd El-Hamid S et al. Genetic variants of neurotransmitter-related genes and miRNAs in Egyptian autistic patients. ScientificWorldJournal 2013; 2013: 670621.

83 Tassone F, Qi L, Zhang W, Hansen RL, Pessah IN, Hertz-Picciotto I. MAOA, DBH, and SLC6A4 variants in CHARGE: a case-control study of autism spectrum disorders. Autism Res 2011; 4: 250–61.

84 Marui T, Hashimoto O, Nanba E, Kato C, Tochigi M, Umekage T et al. Association between the neurofibromatosis-1 (NF1) locus and autism in the Japanese population. Am J Med Genet B Neuropsychiatr Genet 2004; 131B: 43–7.

85 Mbarek O, Marouillat S, Martineau J, Barthélémy C, Müh JP, Andres C. Association study of the NF1 gene and autistic disorder. Am J Med Genet 1999; 88: 729–32.

86 Plank SM, Copeland-Yates SA, Sossey-Alaoui K, Bell JM, Schroer RJ, Skinner C et al. Lack of association of the (AAAT)6 allele of the GXAlu tetranucleotide repeat in intron 27b of the NF1 gene with autism. Am J Med Genet 2001; 105: 404–5.

87 Campbell DB, Sutcliffe JS, Ebert PJ, Militerni R, Bravaccio C, Trillo S et al. A genetic variant that disrupts MET transcription is associated with autism. Proc Natl Acad Sci U S A 2006; 103: 16834–9.

88 Jackson PB, Boccuto L, Skinner C, Collins JS, Neri G, Gurrieri F et al. Further evidence that the rs1858830 C variant in the promoter region of the MET gene is associated with autistic disorder. Autism Res 2009; 2: 232–6.

89 Sousa I, Clark TG, Toma C, Kobayashi K, Choma M, Holt R et al. MET and autism susceptibility: family and case-control studies. Eur J Hum Genet 2009; 17: 749–58.

90 Campbell DB, Li C, Sutcliffe JS, Persico AM, Levitt P. Genetic evidence implicating multiple genes in the MET receptor tyrosine kinase pathway in autism spectrum disorder. Autism Res 2008; 1: 159–68.

91 Thanseem I, Nakamura K, Miyachi T, Toyota T, Yamada S, Tsujii M et al. Further evidence for the role of MET in autism susceptibility. Neurosci Res 2010; 68: 137–41.

92 Zhou X, Xu Y, Wang J, Zhou H, Liu X, Ayub Q et al. Replication of the association of a MET variant with autism in a Chinese Han population. PLoS One 2011; 6: e27428.

93 Wu Y-Y, Chien W-H, Huang Y-S, Gau SS-F, Chen C-H. Lack of evidence to support the glyoxalase 1 gene (GLO1) as a risk gene of autism in Han Chinese patients from Taiwan. Prog Neuropsychopharmacol Biol Psychiatry 2008; 32: 1740–4.

94 Junaid MA, Kowal D, Barua M, Pullarkat PS, Sklower Brooks S, Pullarkat RK. Proteomic studies identified a single nucleotide polymorphism in glyoxalase I as autism susceptibility factor. Am J Med Genet A 2004; 131: 11–7.

95 Kovač J, Podkrajšek KT, Lukšič MM, Battelino T. Weak association of glyoxalase 1 (GLO1) variants with autism spectrum disorder. Eur Child Adolesc Psychiatry 2014. doi:10.1007/s00787-014-0537-8.

96 Sacco R, Papaleo V, Hager J, Rousseau F, Moessner R, Militerni R et al. Case-control and family-based association studies of candidate genes in autistic disorder and its endophenotypes: TPH2 and GLO1. BMC Med Genet 2007; 8: 11.

97 Liu X, Kawamura Y, Shimada T, Otowa T, Koishi S, Sugiyama T et al. Association of the oxytocin receptor (OXTR) gene polymorphisms with autism spectrum disorder (ASD) in the Japanese population. J Hum Genet 2010; 55: 137–41.

98 Jacob S, Brune CW, Carter CS, Leventhal BL, Lord C, Cook EH. Association of the oxytocin receptor gene (OXTR) in Caucasian children and adolescents with autism. Neurosci Lett 2007; 417: 6–9.

99 Tansey KE, Brookes KJ, Hill MJ, Cochrane LE, Gill M, Skuse D et al. Oxytocin receptor (OXTR) does not play a major role in the aetiology of autism: genetic and molecular studies. Neurosci Lett 2010; 474: 163–7.

100 Di Napoli A, Warrier V, Baron-Cohen S, Chakrabarti B. Genetic variation in the oxytocin receptor (OXTR) gene is associated with Asperger Syndrome. Mol Autism 2014; 5: 48.

101 Vourc'h P, Martin I, Marouillat S, Adrien JL, Barthélémy C, Moraine C et al. Molecular analysis of the oligodendrocyte myelin glycoprotein gene in autistic disorder. Neurosci Lett 2003; 338: 115–8.

102 Martin I, Gauthier J, D'Amelio M, Védrine S, Vourc'h P, Rouleau GA et al. Transmission disequilibrium study of an oligodendrocyte and myelin glycoprotein gene allele in 431 families with an autistic proband. Neurosci Res 2007; 59: 426–30.

103 Devlin B, Bennett P, Cook EH, Dawson G, Gonen D, Grigorenko EL et al. No evidence for linkage of liability to autism to HOXA1 in a sample from the CPEA network. Am J Med Genet 2002; 114: 667–72.

104 Collins JS, Schroer RJ, Bird J, Michaelis RC. The HOXA1 A218G Polymorphism and Autism: Lack of Association in White and Black Patients from the South Carolina Autism Project. J Autism Dev Disord 2003; 33: 343–348.

105 Conciatori M, Stodgell CJ, Hyman SL, O'Bara M, Militerni R, Bravaccio C et al. Association between the HOXA1 A218G polymorphism and increased head circumference in patients with autism. Biol Psychiatry 2004; 55: 413–9.

106 Sen B, Sinha S, Ahmed S, Ghosh S, Gangopadhyay PK, Usha R. Lack of association of HOXA1 and HOXB1 variants with autism in the Indian population. Psychiatr Genet 2007; 17: 1.

107 Gallagher L, Hawi Z, Kearney G, Fitzgerald M, Gill M. No association between allelic variants of HOXA1/HOXB1 and autism. Am J Med Genet B Neuropsychiatr Genet 2004; 124B: 64–7.

108 Romano V, Calì F, Mirisola M, Gambino G, D' Anna R, Di Rosa P et al. Lack of association of HOXA1 and HOXB1 mutations and autism in Sicilian (Italian) patients. Mol Psychiatry 2003; 8: 716–7.

109 Talebizadeh Z, Bittel DC, Miles JH, Takahashi N, Wang CH, Kibiryeva N et al. No association between HOXA1 and HOXB1 genes and autism spectrum disorders (ASD). J Med Genet 2002; 39: e70.

110 Li J, Tabor HK, Nguyen L, Gleason C, Lotspeich LJ, Spiker D et al. Lack of association between HoxA1 and HoxB1 gene variants and autism in 110 multiplex families. Am J Med Genet 2002; 114: 24–30.

111 Ingram JL, Stodgell CJ, Hyman SL, Figlewicz DA, Weitkamp LR, Rodier PM. Discovery of allelic variants of HOXA1 and HOXB1: genetic susceptibility to autism spectrum disorders. Teratology 2000; 62: 393–405.

112 Ramoz N, Reichert JG, Corwin TE, Smith CJ, Silverman JM, Hollander E et al. Lack of evidence for association of the serotonin transporter gene SLC6A4 with autism. Biol Psychiatry 2006; 60: 186–91.

113 Devlin B, Cook EH, Coon H, Dawson G, Grigorenko EL, McMahon W et al. Autism and the serotonin transporter: the long and short of it. Mol Psychiatry 2005; 10: 1110–6.

114 Kim S-J, Cox N, Courchesne R, Lord C, Corsello C, Akshoomoff N et al. Transmission disequilibrium mapping at the serotonin transporter gene (SLC6A4) region in autistic disorder. Mol Psychiatry 2002; 7: 278–88.

115 Klauck SM, Poustka F, Benner A, Lesch KP, Poustka A. Serotonin transporter (5-HTT) gene variants associated with autism? Hum Mol Genet 1997; 6: 2233–8.

116 Cook EH, Courchesne R, Lord C, Cox NJ, Yan S, Lincoln A et al. Evidence of linkage between the serotonin transporter and autistic disorder. Mol Psychiatry 1997; 2: 247–50.

117 Conroy J, Meally E, Kearney G, Fitzgerald M, Gill M, Gallagher L. Serotonin transporter gene and autism: a haplotype analysis in an Irish autistic population. Mol Psychiatry 2004; 9: 587–93.

118 Maestrini E, Lai C, Marlow A, Matthews N, Wallace S, Bailey A et al. Serotonin transporter (5-HTT) and gamma-aminobutyric acid receptor subunit beta3 (GABRB3) gene polymorphisms are not associated with autism in the IMGSA families. The International Molecular Genetic Study of Autism Consortium. Am J Med Genet 1999; 88: 492–6.

119 Persico AM, Militerni R, Bravaccio C, Schneider C, Melmed R, Conciatori M et al. Lack of association between serotonin transporter gene promoter variants and autistic disorder in two ethnically distinct samples. Am J Med Genet 2000; 96: 123–7.

120 Tordjman S, Gutknecht L, Carlier M, Spitz E, Antoine C, Slama F et al. Role of the serotonin transporter gene in the behavioral expression of autism. Mol Psychiatry 2001; 6: 434–9.

121 Betancur C, Corbex M, Spielewoy C, Philippe A, Laplanche JL, Launay JM et al. Serotonin transporter gene polymorphisms and hyperserotonemia in autistic disorder. Mol Psychiatry 2002; 7: 67–71.

122 Mulder EJ, Anderson GM, Kema IP, Brugman AM, Ketelaars CEJ, de Bildt A et al. Serotonin transporter intron 2 polymorphism associated with rigid-compulsive behaviors in Dutch individuals with pervasive developmental disorder. Am J Med Genet B Neuropsychiatr Genet 2005; 133B: 93–6.

123 Koishi S, Yamamoto K, Matsumoto H, Koishi S, Enseki Y, Oya A et al. Serotonin transporter gene promoter polymorphism and autism: a family-based genetic association study in Japanese population. Brain Dev 2006; 28: 257–60.

124 Guhathakurta S, Sinha S, Ghosh S, Chatterjee A, Ahmed S, Gangopadhyay PK et al. Population-based association study and contrasting linkage disequilibrium pattern reveal genetic association of SLC6A4 with autism in the Indian population from West Bengal. Brain Res 2008; 1240: 12–21.

125 Wu S, Guo Y, Jia M, Ruan Y, Shuang M, Liu J et al. Lack of evidence for association between the serotonin transporter gene (SLC6A4) polymorphisms and autism in the Chinese trios. Neurosci Lett 2005; 381: 1–5.

126 Yoo HJ, Cho IH, Park M, Yang SY, Kim SA. No Association Study of SLC6A4 Polymorphisms with Korean Autism Spectrum Disorder. Korean J Biol Psychiatry 2009; 16: 121–126.

127 Alarcón M, Abrahams BS, Stone JL, Duvall JA, Perederiy J V, Bomar JM et al. Linkage, association, and gene-expression analyses identify CNTNAP2 as an autism-susceptibility gene. Am J Hum Genet 2008; 82: 150–9.

128 Anderson BM, Schnetz-Boutaud N, Bartlett J, Wright HH, Abramson RK, Cuccaro ML et al. Examination of association to autism of common genetic variationin genes related to dopamine. Autism Res 2008; 1: 364–9.

129 Cheng L, Ge Q, Sun B, Yu P, Ke X, Lu Z. Polyacrylamide gel-based microarray: a novel method applied to the association Study between the polymorphisms of BDNF gene and autism. J Biomed Nanotechnol 2009; 5: 542–50.

130 Egawa J, Watanabe Y, Nunokawa A, Endo T, Kaneko N, Tamura R et al. A detailed association analysis between the tryptophan hydroxylase 2 (TPH2) gene and autism spectrum disorders in a Japanese population. Psychiatry Res 2012; 196: 320–2.

131 Gaita L, Manzi B, Sacco R, Lintas C, Altieri L, Lombardi F et al. Decreased serum arylesterase activity in autism spectrum disorders. Psychiatry Res 2010; 180: 105–13.

132 Hutcheson HB, Olson LM, Bradford Y, Folstein SE, Santangelo SL, Sutcliffe JS et al. Examination of NRCAM, LRRN3, KIAA0716, and LAMB1 as autism candidate genes. BMC Med Genet 2004; 5: 12.

133 Kelemenova S, Schmidtova E, Ficek A, Celec P, Kubranska A, Ostatnikova D. Polymorphisms of candidate genes in Slovak autistic patients. Psychiatr Genet 2010; 20: 137–9.

134 Mei H, Cuccaro ML, Martin ER. Multifactor dimensionality reduction-phenomics: a novel method to capture genetic heterogeneity with use of phenotypic variables. Am J Hum Genet 2007; 81: 1251–61.

135 Petit E, Hérault J, Martineau J, Perrot A, Barthélémy C, Hameury L et al. Association study with two markers of a human homeogene in infantile autism. J Med Genet 1995; 32: 269–74.

136 Rabionet R, McCauley JL, Jaworski JM, Ashley-Koch AE, Martin ER, Sutcliffe JS et al. Lack of association between autism and SLC25A12. Am J Psychiatry 2006; 163: 929–31.

137 Rehnström K, Ylisaukko-Oja T, Vanhala R, von Wendt L, Peltonen L, Hovatta I. No association between common variants in glyoxalase 1 and autism spectrum disorders. Am J Med Genet B Neuropsychiatr Genet 2008; 147B: 124–7.

138 Serajee FJ, Nabi R, Zhong H, Huq M. Polymorphisms in xenobiotic metabolism genes and autism. J Child Neurol 2004; 19: 413–7.

139 Veatch OJ, Pendergast JS, Allen MJ, Leu RM, Johnson CH, Elsea SH et al. Genetic Variation in Melatonin Pathway Enzymes in Children with Autism Spectrum Disorder and Comorbid Sleep Onset Delay. J Autism Dev Disord 2014. doi:10.1007/s10803-014-2197-4.

140 Weiss LA, Ober C, Cook EH. ITGB3 shows genetic and expression interaction with SLC6A4. Hum Genet 2006; 120: 93–100.

141 Xu X-M, Ding M, Pang H, Xing J-X, Xuan J-F, Wang B-J. [Genetic polymorphisms of SNP loci in the 5’ and 3' region of TPH2 gene in Northern Chinese Han population]. Fa Yi Xue Za Zhi 2013; 29: 21–4.

142 McCauley JL, Olson LM, Dowd M, Amin T, Steele A, Blakely RD et al. Linkage and association analysis at the serotonin transporter (SLC6A4) locus in a rigid-compulsive subset of autism. Am J Med Genet B Neuropsychiatr Genet 2004; 127B: 104–12.

143 Arking DE, Cutler DJ, Brune CW, Teslovich TM, West K, Ikeda M et al. A common genetic variant in the neurexin superfamily member CNTNAP2 increases familial risk of autism. Am J Hum Genet 2008; 82: 160–4.

144. Ashley-Koch AE, Jaworski J, Ma de Q, Mei H, Ritchie MD, Skaar DA et al. Investigation of potential gene-gene interactions between APOE and RELN contributing to autism risk. Psychiatr Genet. 2007 Aug;17(4):221-6.

Supplementary Figures 1 – 8: Forest plots of most significant SNPs

Figure 1: Forest plot for rs7794745 (*CNTNAP2*)

Study name	Subgroup within study	Odds ratio	Lower limit	Upper limit	Z-Value	p-Value
Li 2010	TDT	0.919	0.677	1.247	-0.545	0.586
Toma 2013	CC	0.756	0.567	1.006	-1.917	0.055
Sampath 2013 NIMH	TDT	0.841	0.757	0.935	-3.210	0.001
Sampath 2013 SSC	TDT	0.944	0.854	1.042	-1.141	0.254
		0.887	0.828	0.950	-3.445	0.001

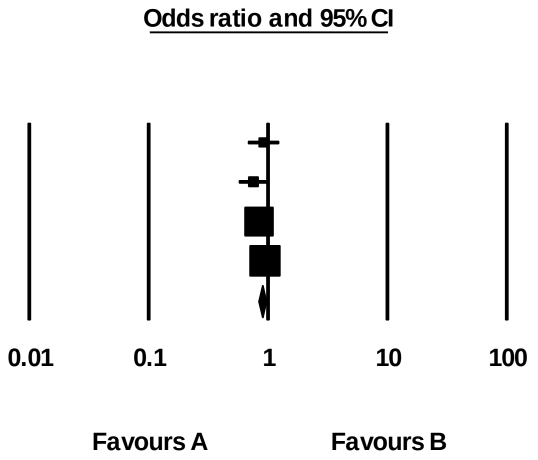

Figure 2: Forest plot for rs167771 (*DRD3*)

Statistics for each study

	Odds ratio	Lower limit	Upper limit	Z-Value	p-Value
Krom 2009 A	1.884	1.348	2.633	3.708	0.000
Krom 2009 B	2.247	1.372	3.680	3.218	0.001
Toma 2013	0.700	0.282	1.734	-0.771	0.441
	1.822	1.398	2.375	4.438	0.000

Odds ratio and 95% CI

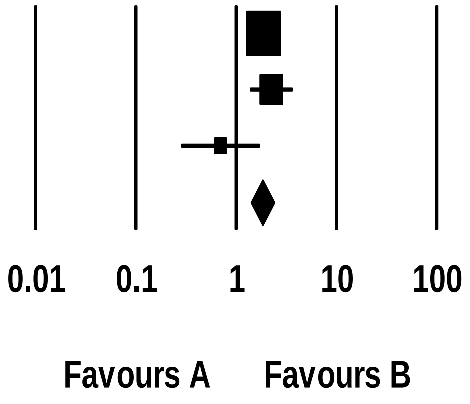

Figure 3: Forest plot for rs362691 (*RELN*)

Study name	Odds ratio	Lower limit	Upper limit	Z-Value	p-Value
Sharma 2013 (Black)	1.060	0.372	3.023	0.109	0.913
Sharma 2013 (White)	0.590	0.220	1.581	-1.049	0.294
Sharma 2013 (Mixed)	0.740	0.311	1.758	-0.682	0.495
He 2011	1.405	0.793	2.490	1.164	0.244
Dutta 2008	0.810	0.432	1.518	-0.658	0.511
Li 2008	0.850	0.773	0.934	-3.374	0.001
Bonora 2003	0.800	0.413	1.550	-0.661	0.509
Serajee 2006	0.520	0.360	0.751	-3.492	0.000
	0.832	0.763	0.908	-4.112	0.000

Statistics for each study

Odds ratio and 95% CI

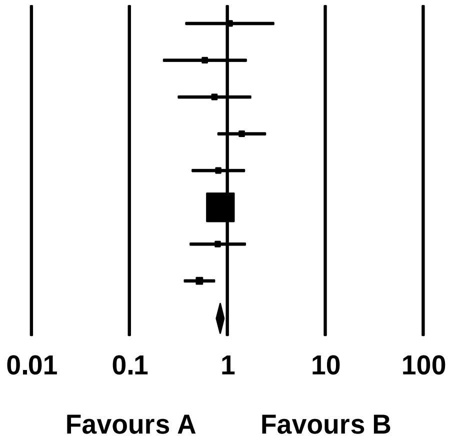

Figure 4: Forest plot for rs2268491 (*OXTR*)

Study name	Odds ratio	Lower limit	Upper limit	Z-Value	p-Value
Liu et al., 2010	1.405	1.112	1.775	2.850	0.004
Tansey et al., 2010 Irish	1.410	0.860	2.311	1.363	0.173
Tansey et al., 2010 Portuguese	1.020	0.678	1.535	0.095	0.924
Tansey et al., 2010 UCL	1.250	0.588	2.659	0.579	0.562
	1.310	1.092	1.572	2.906	0.004

Statistics for each study

Odds ratio and 95%CI

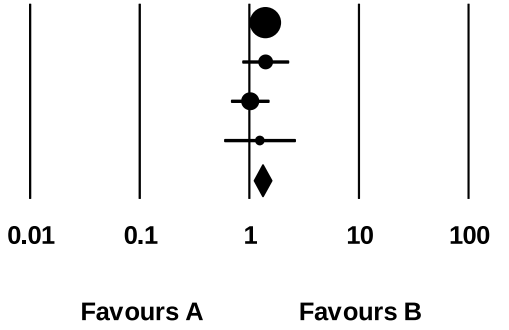

Figure 5: Forest plot for rs2292813 (*SLC25A12*)

Study name	Subgroup within study	Outcome	Odds ratio	Lower limit	Upper limit	Z-Value	p-Value
Ramoz 2004	Egyptian	TDT	1.459	1.107	1.922	2.682	0.007
Segurado 2005	Irish	TDT	1.889	1.067	3.344	2.182	0.029
Blasi 2006	Caucasian	TDT	1.280	0.884	1.853	1.308	0.191
Chien 2010	Chinese	Case-control	1.293	0.950	1.758	1.636	0.102
Palmieri 2010	Italian	Case-control	1.182	0.472	2.958	0.357	0.721
Prandini 2012	Italian	TDT	0.710	0.213	2.364	-0.558	0.577
			1.373	1.162	1.622	3.722	0.000

(Statistics for each study)

Odds ratio and 95% CI

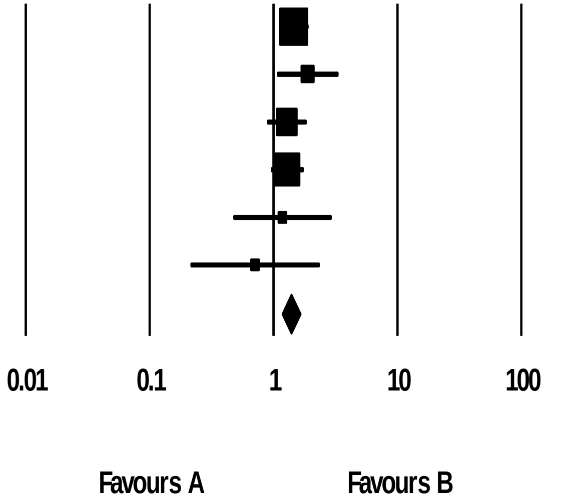

Figure 6: Forest plot for rs2056202 (*SLC25A12*)

Study name	Odds ratio	Lower limit	Upper limit	Z-Value	p-Value
	Statistics for each study				
Ramoz 2004	1.394	1.119	1.736	2.968	0.003
Segurado 2005	1.840	1.131	2.994	2.454	0.014
Blasi 2006	1.125	0.832	1.520	0.767	0.443
Correira 2006	1.031	0.532	1.998	0.090	0.928
Chakrabarti 2009	1.112	0.739	1.672	0.510	0.610
Chien 2010	1.093	0.824	1.450	0.619	0.536
Palmieri 2010	1.361	0.584	3.170	0.713	0.476
Durdiakova 2014	0.760	0.393	1.470	-0.815	0.415
	1.227	1.079	1.396	3.123	0.002

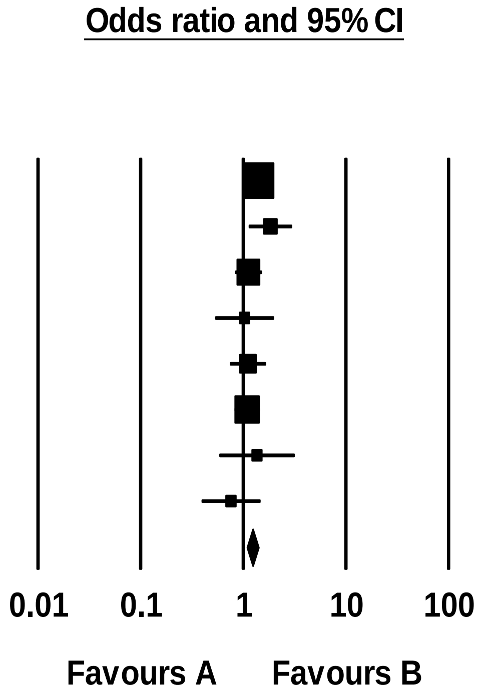

Figure 7: Forest plot for rs1801133 (*MTHFR*)

Study name	Odds ratio	Lower limit	Upper limit	Z-Value	p-Value
Boris 2004	2.252	1.811	2.799	7.307	0.000
James 2006	1.243	0.962	1.606	1.666	0.096
Mohammad 2009	2.790	1.579	4.928	3.535	0.000
Pasca 2009	1.179	0.553	2.510	0.426	0.670
dos Santo 2010	1.150	0.789	1.676	0.727	0.467
Liu 2011	1.148	0.980	1.343	1.714	0.086
Schmidt 2011	0.853	0.649	1.121	-1.140	0.254
Guo 2012	1.303	0.961	1.767	1.703	0.089
Divyakolu 2013	3.632	1.543	8.547	2.953	0.003
Park 2014	0.966	0.772	1.208	-0.307	0.759
	1.370	1.079	1.739	2.589	0.010

Odds ratio and 95% CI

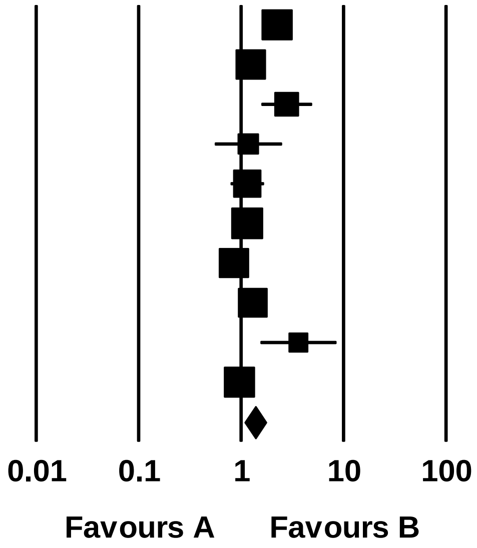

Figure 8: Forest plot for rs1861972 (*EN2*)

Meta Analysis

Study name	Subgroup within study	Odds ratio	Lower limit	Upper limit	Z-Value	p-Value
Yang 2008	Case-control	1.661	1.045	2.641	2.146	0.032
Yang 2010	Case-control	1.476	0.940	2.319	1.691	0.091
Benayed 2005 AGRE II	TDT	1.082	0.960	1.220	1.295	0.195
Benayed 2005 NIMH	TDT	1.106	0.917	1.335	1.052	0.293
Gharani 2004	TDT	1.826	1.241	2.686	3.056	0.002
Chakrabarti 2009	Case-control	0.839	0.619	1.136	-1.136	0.256
Warrier 2014	Case-control	1.118	0.807	1.549	0.670	0.503
Prandini 2012	TDT	1.136	0.788	1.639	0.684	0.494
		1.125	1.035	1.224	2.758	0.006

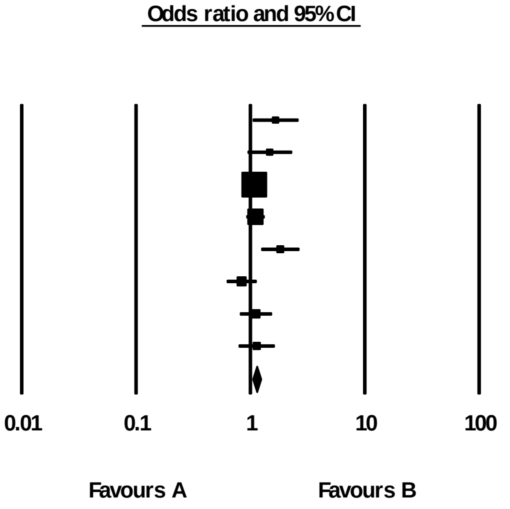

Supplementary Figures 9 – 15: Significant subgroup analyses

Figure 9: STin2 VNTR (*SLC6A4*), Caucasian only

Study name	Subgroup within study	Odds ratio	Lower limit	Upper limit	Z-Value	p-Value
		Statistics for each study				
Cook 1997	Caucasian	1.650	0.857	3.177	1.498	0.134
Klauk 1997	Caucasian	1.400	0.725	2.703	1.003	0.316
Maestrini 1999	Caucasian	0.910	0.499	1.660	-0.307	0.759
Kim 2002	Caucasian	3.380	1.519	7.522	2.984	0.003
		1.492	1.068	2.083	2.347	0.019

Odds ratio and 95% CI

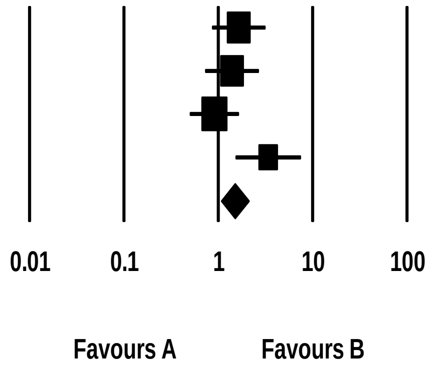

Figure 10: rs362691 (*RELN*), Case-control only

Study name	Method	Ancestry	Odds ratio	Lower limit	Upper limit	Z-Value	p-Value
			Statistics for each study				
Sharma 2013 (Black)	Case control	South African	1.060	0.372	3.023	0.109	0.913
Sharma 2013 (White)	Case control	South African	0.590	0.220	1.581	-1.049	0.294
Sharma 2013 (Mixed)	Case control	South African	0.740	0.311	1.758	-0.682	0.495
He 2011	Case control	Chinese (Hans)	1.405	0.793	2.490	1.164	0.244
Dutta 2008	Case control	Indian	0.810	0.432	1.518	-0.658	0.511
Li 2008	Case control	Chinese (Hans)	0.850	0.773	0.934	-3.374	0.001
			0.857	0.783	0.939	-3.318	0.001

Odds ratio and 95% CI

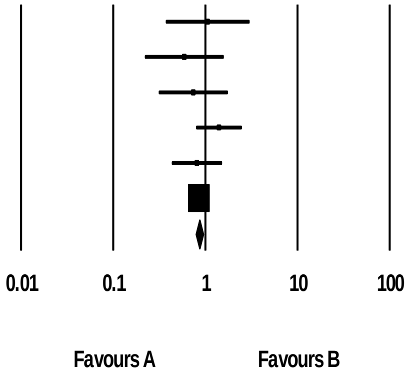

Figure 11: rs2292813 (*SLC25A12*), TDT only

Group by Outcome	Study name	Subgroup within study	Outcome	Odds ratio	Lower limit	Upper limit	Z-Value	p-Value
				Statistics for each study				
TDT	Ramoz 2004	Egyptian	TDT	1.459	1.107	1.922	2.682	0.007
TDT	Segurado 2005	Irish	TDT	1.889	1.067	3.344	2.182	0.029
TDT	Blasi 2006	Caucasian	TDT	1.280	0.884	1.853	1.308	0.191
TDT	Prandini 2012	Italian	TDT	0.710	0.213	2.364	-0.558	0.577
TDT				1.419	1.158	1.740	3.377	0.001

Odds ratio and 95% CI

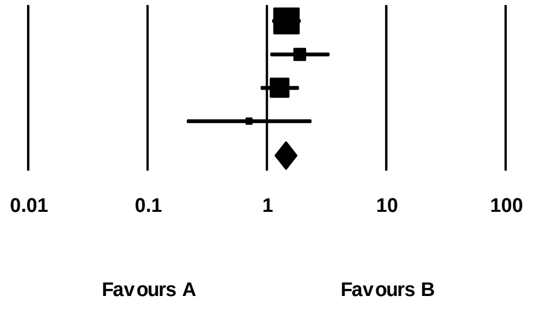

Figure 12: rs2056202 (*SLC25A12*), TDT only

Meta Analysis

Study name	Ancestry	Method	Odds ratio	Lower limit	Upper limit	Z-Value	p-Value
			Statistics for each study				
Ramoz 2004	Egyptian	TDT	1.394	1.119	1.736	2.968	0.003
Segurado 2005	Irish	TDT	1.840	1.131	2.994	2.454	0.014
Blasi 2006	Caucasian	TDT	1.125	0.832	1.520	0.767	0.443
Chakrabarti 2009	Caucasian	TDT	1.112	0.739	1.672	0.510	0.610
Durdiakova 2014	Caucasian	TDT	0.760	0.393	1.470	-0.815	0.415
			1.275	1.097	1.482	3.173	0.002

Odds ratio and 95% CI

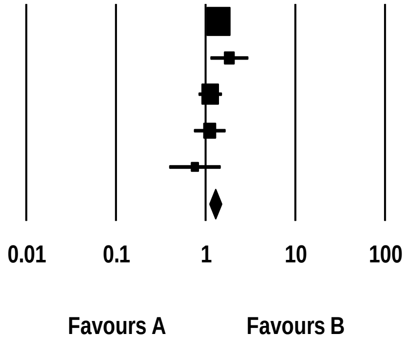

Figure 13: rs1861973 (*EN2*), TDT only

Study name	Subgroup within study	Statistics for each study				
		Odds ratio	Lower limit	Upper limit	Z-Value	p-Value
Sen 2010	TDT	0.568	0.333	0.970	-2.073	0.038
Gharani 2004	TDT	0.715	0.550	0.929	-2.507	0.012
Benayed 2005 AGRE II	TDT	0.916	0.811	1.034	-1.419	0.156
Benayed 2005 NIMH	TDT	0.893	0.741	1.076	-1.189	0.234
		0.869	0.792	0.954	-2.942	0.003

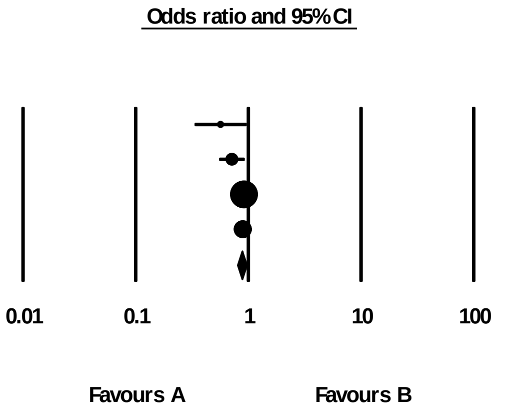

Figure 14: rs1861973 (*EN2*), Caucasian only

Study name	Subgroup within study	Odds ratio	Lower limit	Upper limit	Z-Value	p-Value
Gharani 2004	Caucasian	0.715	0.550	0.929	-2.507	0.012
Benayed 2005 AGRE II	Caucasian	0.916	0.811	1.034	-1.419	0.156
Benayed 2005 NIMH	Caucasian	0.893	0.741	1.076	-1.189	0.234
		0.881	0.801	0.969	-2.620	0.009

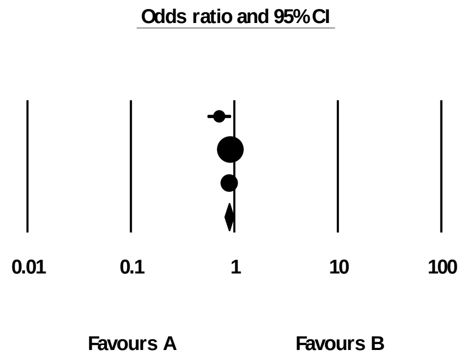

Figure 15: rs1861972 (*EN2*), TDT only

Meta Analysis

Study name	Subgroup within study	Odds ratio	Lower limit	Upper limit	Z-Value	p-Value
Benayed 2005 AGRE II	TDT	1.082	0.960	1.220	1.295	0.195
Benayed 2005 NIMH	TDT	1.106	0.917	1.335	1.052	0.293
Gharani 2004	TDT	1.826	1.241	2.686	3.056	0.002
Prandini 2012	TDT	1.136	0.788	1.639	0.684	0.494
		1.126	1.025	1.238	2.472	0.013

(Statistics for each study)

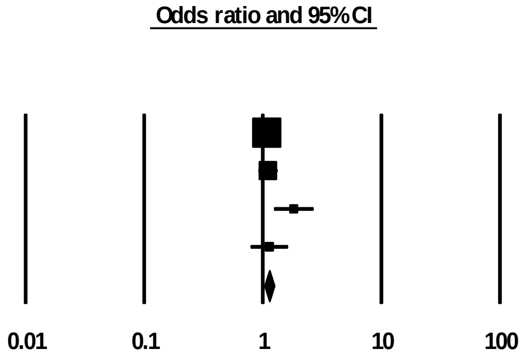

Supplementary Figures 16 – 20: Sensitivity analyses forest plots

Figure 16: Sensitivity analysis for rs4446909 (*ASMT*)

Study name	Ancestry	Statistics with study removed				
		Point	Lower limit	Upper limit	Z-Value	p-Value
Melke 2008	Mixed	1.120	0.955	1.314	1.391	0.164
Toma 2007 Finnish	Caucasian	1.194	1.033	1.381	2.394	0.017
Toma 2007 Italian	Caucasian	1.201	1.037	1.390	2.450	0.014
Toma 2007 IMGSAC	Caucasian	1.213	1.038	1.418	2.430	0.015
Wang 2013	Chinese	1.272	1.051	1.539	2.468	0.014
		1.195	1.038	1.375	2.479	0.013

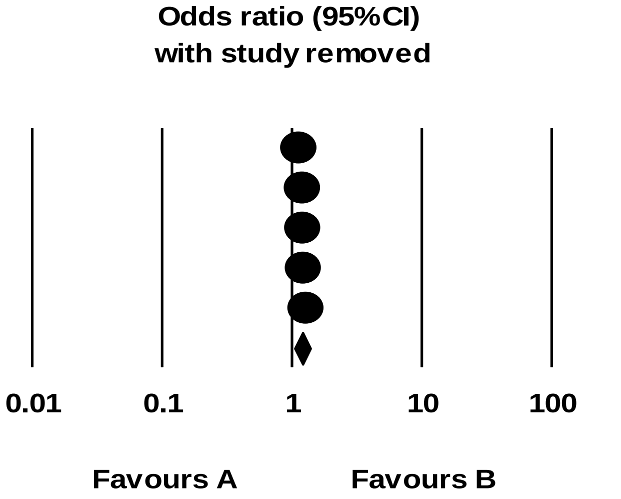

Figure 17: Sensitivity analysis for rs736707 (*RELN*)

Meta Analysis

Study name	Ancestry	Method	Statistics with study removed				
			Point	Lower limit	Upper limit	Z-Value	p-Value
Sharma 2013 (Black)	South African	Case control	1.297	1.036	1.623	2.268	0.023
Sharma 2013 (White)	South African	Case control	1.266	1.014	1.580	2.084	0.037
Sharma 2013 (Mixed)	South African	Case control	1.204	0.978	1.483	1.749	0.080
He 2011	Chinese (Hans)	Case control	1.363	1.140	1.629	3.402	0.001
Dutta 2008	Indian	Case control	1.262	1.002	1.589	1.979	0.048
Li 2008	Chinese (Hans)	Case control	1.267	0.956	1.679	1.644	0.100
Serajee 2006	Caucasian	TDT	1.187	0.953	1.479	1.527	0.127
Chakrabarti 2009	Caucasian	Case control	1.310	1.038	1.652	2.277	0.023
Warrier 2014	Caucasian	Case control	1.302	1.034	1.640	2.248	0.025
			1.269	1.030	1.563	2.235	0.025

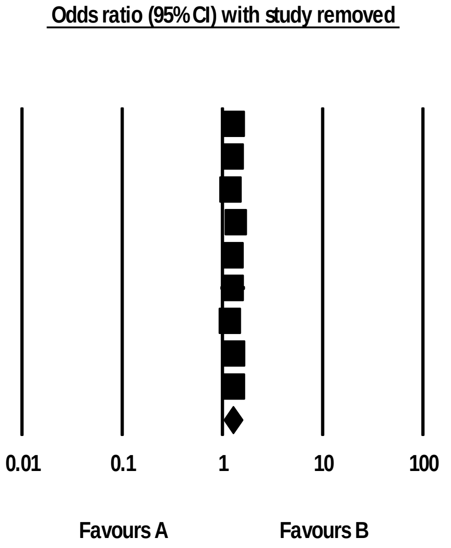

Figure 18: Sensitivity analysis for rs1801133 (*MTHFR*)

Meta Analysis

Study name	Statistics with study removed				
	Point	Lower limit	Upper limit	Z-Value	p-Value
Boris 2004	1.229	1.019	1.483	2.153	0.031
James 2006	1.397	1.064	1.835	2.408	0.016
Mohammad 2009	1.288	1.017	1.632	2.098	0.036
Pasca 2009	1.385	1.079	1.777	2.558	0.011
dos Santo 2010	1.402	1.081	1.820	2.543	0.011
Liu 2011	1.422	1.064	1.900	2.382	0.017
Schmidt 2011	1.455	1.135	1.865	2.958	0.003
Guo 2012	1.386	1.062	1.809	2.399	0.016
Divyakolu 2013	1.301	1.028	1.646	2.190	0.029
Park 2014	1.441	1.110	1.872	2.741	0.006
	1.370	1.079	1.739	2.589	0.010

Odds ratio (95% CI) with study removed

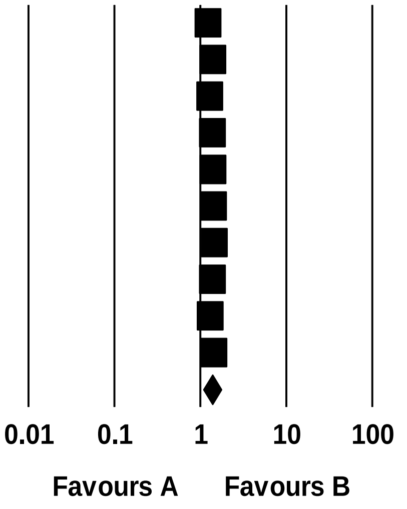

Figure 19: Sensitivity analysis for rs2056202 (*SLC25A12*)

Meta Analysis

Study name	Statistics with study removed				
	Point	Lower limit	Upper limit	Z-Value	p-Value
Ramoz 2004	1.148	0.980	1.346	1.708	0.088
Segurado 2005	1.191	1.042	1.360	2.566	0.010
Blasi 2006	1.251	1.085	1.442	3.091	0.002
Correira 2006	1.236	1.084	1.409	3.165	0.002
Chakrabarti 2009	1.241	1.084	1.421	3.121	0.002
Chien 2010	1.265	1.095	1.461	3.191	0.001
Palmieri 2010	1.224	1.075	1.394	3.050	0.002
Durdiakova 2014	1.251	1.097	1.426	3.345	0.001
	1.227	1.079	1.396	3.123	0.002

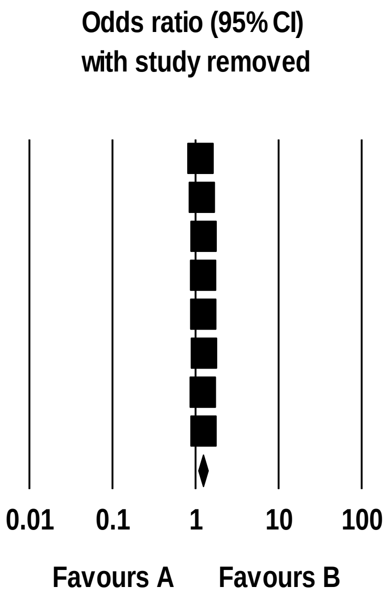

Figure 20: Sensitivity analysis for rs1861972 (*EN2*)

Meta Analysis

Study name	Statistics with study removed					Odds ratio (95% CI) with study removed
	Point	Lower limit	Upper limit	Z-Value	p-Value	
Yang 2008	1.147	0.978	1.344	1.690	0.091	
Yang 2010	1.163	0.982	1.377	1.750	0.080	
Benayed 2005 AGRE II	1.242	0.995	1.550	1.915	0.055	
Benayed 2005 NIMH	1.228	0.993	1.518	1.898	0.058	
Gharani 2004	1.112	0.975	1.268	1.579	0.114	
Chakrabarti 2009	1.244	1.060	1.460	2.674	0.007	
Warrier 2014	1.207	1.002	1.455	1.980	0.048	
	1.187	1.011	1.393	2.092	0.036	

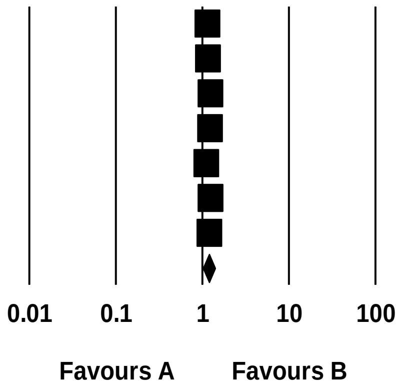

Details of data from our lab

Cohort 1: Cohort 1 consists of 349 controls (143 males and 206 females) without an ASC diagnosis and were recruited using an advertisement. The mean AQ score for this cohort was 16.43 (range: 3–36, mean score for males: 18.01, mean score for females: 15.33). There were 174 cases (140 males and 34 females). Cases were diagnosed with AS by independent clinicians based on DSM-IV or ICD 10 criteria. The following SNPs used included in the study were genotyped an analysed previously [1]: rs37356353 and rs1861972 in *EN2*; rs6265 in *BDNF*, rs10951145 in *HOXA1*; rs237885 and rs2228485 in *OXTR*. Additionally, for this study, we genotyped the following SNPs: rs4717806 in *STX1A*; rs736707 in *RELN*, rs2056202 in SLC25A12; rs53576, rs2254298, rs2268493, rs2268490, rs237894, and rs2301261 in *OXTR*.

Cohort 2: Cohort 2 consists of 118 cases and 412 controls. The controls (185 males, 227 females) had an AQ score below 24. The mean AQ score was 14.9 (range: 2-23, mean score for males: 16.0, mean score for females: 13.9). There were 118 cases (74 males, and 44 females). Cases were diagnosed with AS by independent clinicians based on DSM-IV or ICD 10 criteria. Select SNPs in three genes, namely OXTR, SLC25A12, GABRB3, and STX1A were genotyped, analysed and reported previously [2,3,4,5]. SNPs from these genes analysed in this study have been referenced accordingly. In addition, we also genotyped rs1861972 in *EN2* and rs736707 in *RELN*.

All participants reported Caucasian ancestry for atleast three generations.DNA was extracted from buccal swabs. Genotyping was performed using TaqMan® SNP genotyping assays, Applied Biosystems Inc., CA. No SNP showed a significant deviation from HWE. Allelic association study was performed using Plink v1.07 [6].

Previous meta-analyses

Five genes investigated in our study have been previously investigated in other meta-analyses. These are: *OXTR*, *RELN*, *HOXA1*, *MTHFR*, and *SLC6A4*. *HOXB1* was previously analysed using meta-analysis and was not re-investigated in our study as there was no new data to include. Our study differs from that of LoParo and Waldman [7], who carried out a meta-analysis of *OXTR* and ASC, as they included FBAT studies in their analyses. We excluded studies that used FBAT as FBAT does not report effect sizes. However, we included three additional cohorts, unpublished genotype data from two cohorts from our lab, and a third cohort studied by Nyffeler and colleagues [8]. Of the three variants significant in the previous study, rs237887 andrs2268491 were significant in our study. We did not have enough data to test the third significant variant (rs7632287).

A previous ASC and *RELN* meta-analysis [9] investigated three variants (rs736707, rs362691, and the GGC repeat), with only rs362691 giving a statistically significant P-value. In our study, we re-investigated the first two variants using data from additional cohorts. rs736707 was nominally significant and rs362691 was significant in our study. We did not identify any additional data for the GGC repeat and hence did not investigate it in our study. Additionally, we identified a fourth variant in the *RELN* gene, rs2073559, which was not investigated by the previous meta-analysis. This variant was not significant in our study.

We analysed both the variants investigated in a previous meta-analysis [10], of *MTHFR* and ASC, including data from two additional studies for rs1801133 and one additional study for rs1801131. The results were similar to the previous results obtained. rs1801133 was significant whereas rs1801131 was not. While the previous study stratified based on folate fortification, we did not conduct these analyses due to insufficient data on folate fortification.

We re-investigated rs10951154 in *HOXA1* which was investigated in an earlier meta-analysis [11]. We included data from the Chakrabarti 2009 cohort in our study, which was not included in the earlier study. While the previous study carried out analyses stratified by ethnicity, they

did not stratify the data based on study methodology, differing from our study. We did not identify any additional data from the *HOXB1* variant, rs72338773, investigated in the previous study [11] and hence did not re-investigate that variant.

Finally, *SLC6A4* has been investigated for ASC using meta-analysis in an earlier study [12]. We extend their work for 5-HTTLPR by using additional data and investigate two additional variants (rs2020936 and rs2020942) in our study. We did not identify any additional data for STin2 VNTR and hence did not re-investigate it.

References

1. Chakrabarti B, Dudbridge F, Kent L, Wheelwright S, Hill-Cawthorne G, Allison C, et al. Genes related to sex steroids, neural growth, and social-emotional behavior are associated with autistic traits, empathy, and Asperger syndrome. Autism Res. 2009; 2: 157-77.

2. Di Napoli A, Warrier V, Baron-Cohen S, Chakrabarti B. Genetic variation in the oxytocin receptor (OXTR) gene is associated with Asperger Syndrome. Mol Autism. 2014; 5:48.

3. Durdiaková J, Warrier V, Baron-Cohen S, Chakrabarti B. Single nucleotide polymorphism rs6716901 in SLC25A12 gene is associated with Asperger syndrome. Mol Autism. 2014; 5:25

4. Warrier V, Baron-Cohen S, Chakrabarti B. Genetic variation in GABRB3 is associated with Asperger syndrome and multiple endophenotypes relevant to autism. Mol Autism. 2013 ; 4: 48

5. Durdiaková J, Warrier V, Banerjee-Basu S, Baron-Cohen S, Chakrabarti B. STX1A and Asperger syndrome: a replication study. Mol Autism. 2014; 5:14

6. Purcell S, Neale B, Todd-Brown K, Thomas L, Ferreira MAR, Bender D, Maller J, Sklar P, de Bakker PIW, Daly MJ, Sham PC: PLINK: a tool set for whole-genome association and population-based linkage analyses. Am J Hum Genet. 2007; 81:559-575.

7. LoParo D, Waldman ID. The oxytocin receptor gene (OXTR) is associated with autism spectrum disorder: a meta-analysis. *Mol Psychiatry* 2014 [Epub ahead of print]

8. Nyffeler J, Walitza S, Bobrowski E, Gundelfinger R, Grünblatt E. Association study in siblings and case-controls of serotonin- and oxytocin-related genes with high functioning autism. *J Mol Psychiatry* 2014; **2:** 1

9. Wang Z, Hong Y, Zou L, Zhong R, Zhu B, Shen N *et al*. Reelin gene variants and risk of autism spectrum disorders: an integrated meta-analysis. *Am J Med Genet B Neuropsychiatr Genet* 2014; **165B:** 192-200.

10. Pu D, Shen Y, Wu J. Association between MTHFR gene polymorphisms and the risk of autism spectrum disorders: a meta-analysis. *Autism Res* 2013; **6:** 384-92.

11. Song RR, Zou L, Zhong R, Zheng XW, Zhu BB, Chen W *et al.* An integrated meta-analysis of two variants in HOXA1/HOXB1 and their effect on the risk of autism spectrum disorders. *PLoS One* 2011; **6:** e25603.

12. Huang CH, Santangelo SL. Autism and serotonin transporter gene polymorphisms: a systematic review and meta-analysis. *Am J Med Genet B Neuropsychiatr Genet* 2008;**147B:** 903-13.
